# Direct Photochemical C–H Carboxylation of Aromatic Diamines with CO_2_ under Electron-Donor- and Base-free Conditions

**DOI:** 10.1038/s41598-018-33060-3

**Published:** 2018-10-02

**Authors:** Takeshi Matsumoto, Daiki Uchijo, Takuji Koike, Ryoya Namiki, Ho-Chol Chang

**Affiliations:** 1 Department of Applied Chemistry, Faculty of Science and Engineering, Chuo University, 1-13-27 Kasuga, Bunkyo-ku, Tokyo 112-8551 Japan; 2 Precursory Research for Embryonic Science and Technology (PRESTO), Japan Science and Technology Agency (JST), 4-1-8 Honcho, Kawaguchi, Saitama 332-0012 Japan

## Abstract

We report the photochemical carboxylation of *o*-phenylenedimamine in the absence of a base and an electron donor under an atmosphere of CO_2_, which afforded 2,3-diaminobenzoic acid (DBA) in 28% synthetic yield and 0.22% quantum yield (*Φ*(%)). The synthetic yield of DBA in this reaction increased to 58% (*Φ*(%) = 0.47) in the presence of Fe(II). The photochemical reaction described in this work provides an effective strategy to use light as the driving force for the direct carboxylation of organic molecules by CO_2_.

## Introduction

Owing to the ever-increasing desire to use carbon dioxide (CO_2_) as an C_1_ feedstock^1–6^, the development of heterogeneous^5,7^ and homogeneous^4,8–21^ catalysts for the transformation of CO_2_ into reduced C_1_ compounds^8,9,17–21^ or CO_2_-containing organic compounds has received considerable attention^10–16^. In the former case, several thermal hydrogenation^8,9^, electro-^17–19^, and photo-catalytic systems^17,18,20,21^ that produce reduced C_1_ chemicals, e.g., CH_3_OH^22–24^, CO^25–30^, HCO_2_H^31–33^, and HCO_2_^−^^34–36^ have been investigated. In the latter case, carboxylic acids are formed, which represent a central motif in a variety of synthetically important chemicals^37–50^.

Recently, the direct carboxylation of aryl rings has garnered considerable attention. For example, Nolan *et al*. reported the [M(IPr)(OH)] (M = Cu^I^ or Au^I^, IPr = 1.3-bis(diisopropyl)phenylimidazol-2-ylidene)-catalyzed carboxylation of halogenated benzene using KOH or CsOH as a base under an atmosphere of CO_2_^42,43^. Iwasawa *et al*. also reported the Pd(OAc)_2_-catalyzed photochemical carboxylation of aryl halides using [Ir(ppy)_2_(dtbpy)][PF_6_] (*λ*_ex_ = 425 nm; dtbpy = 4,4′-di-*tert*-butyl-2,2′-bipyridyl) as the photosensitizer and *i*Pr_2_NEt as a sacrificial electron donor under CO_2_^47^. In these reports, the presence of a base and/or electron donor is necessary for the thermal or photochemical reaction and the incorporation of CO_2_. However, obtaining carboxylic acids directly from CO_2_ in the absence of an electron donor and base would be more attractive in terms of atom- and step-economy.

**Figure 1:**
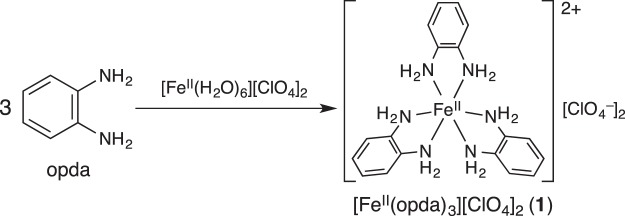
Synthesis of **1** from opda and [Fe^II^(H_2_O)_6_][ClO_4_]_2_.

We have recently reported the photochemical hydrogen evolution (PHE) from the Fe(II) complex [Fe^II^(opda)_3_][ClO_4_]_2_ (**1**; opda = *o*-phenylenediamine) (Fig. 1) under N_2_^51^, wherein opda acts as a photo-responsive proton/electron pool that forms the partially oxidized *semi*-benzoquinodiimine (s-bqdi) or *o-*benzoquinodiimine ligands. The photochemical reaction of **1** under N_2_ inspired us to investigate that under CO_2_. Herein, we report the first example for a direct photochemical carboxylation of C–H bonds in aromatic diamines with CO_2_ in the absence of an electron donor and base.

## Results

### Photochemical hydrogen evolution reactions from opda and 1 under N_2_ or CO_2_

**Figure 2:**
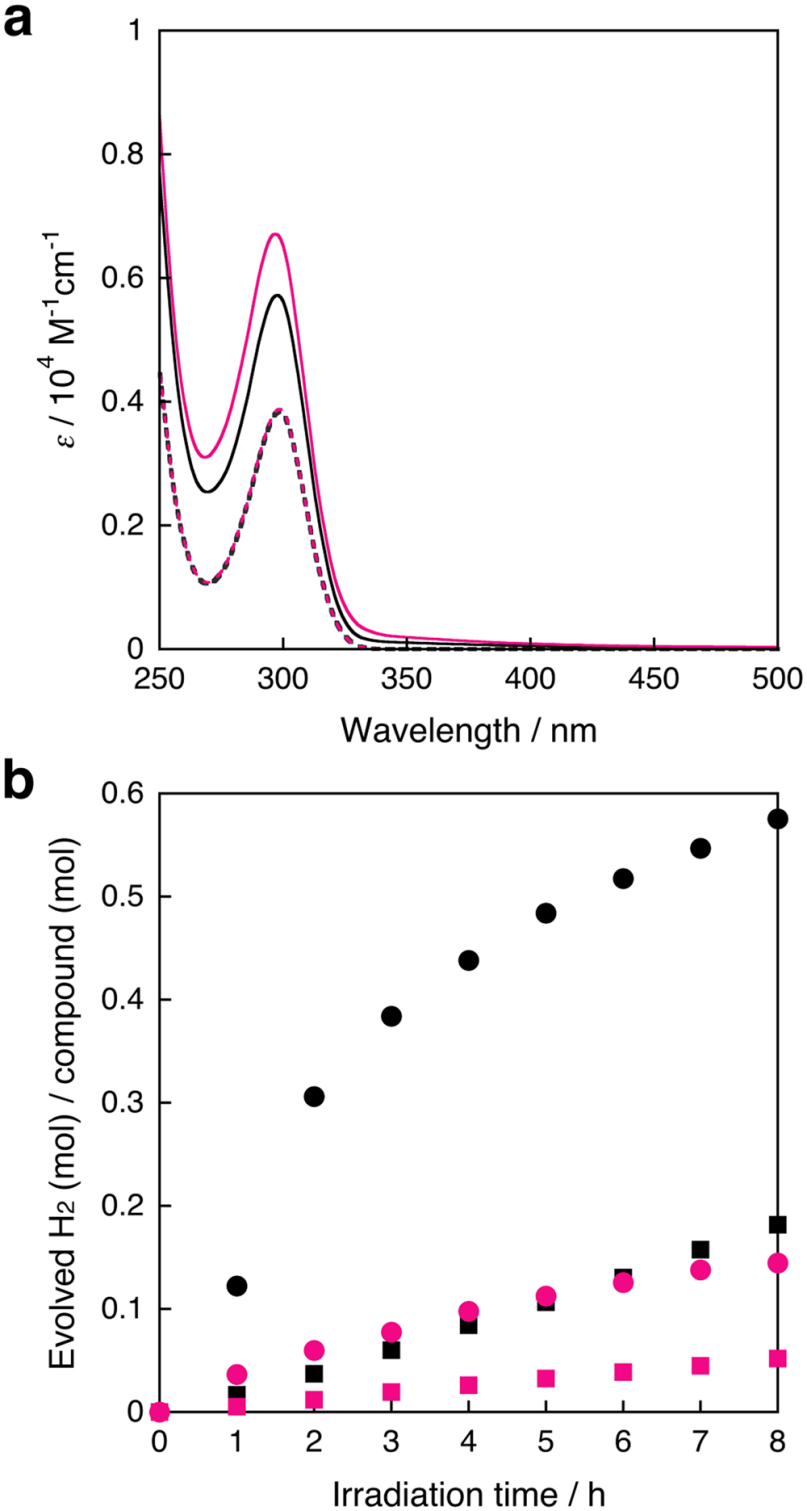
(**a**) UV-Vis spectra of opda (6.0 × 10^−3^ M) under N_2_ (···) or CO_2_ (), and **1** (2.0 × 10^−3^ M) under N_2_ (—) or CO_2_ () in THF at room temperature. (**b**) Time-course plots of the amount of photochemically evolved hydrogen from a) opda (6.0 × 10^−3^ M; 1.2 × 10^−5^ mol/4 mL; *λ*_ex_ = 300 ± 10 nm; 63.5–66.9 mW) under N_2_ (▪) or CO_2_ (), and **1** (2.0 × 10^−3^ M; 8.0 × 10^−6^ mol/4 mL; *λ*_ex_ = 300 ± 10 nm; 63.9–66.9 mW) under N_2_ (•) or CO_2_ () in THF.

The UV-Vis spectra of opda and **1** in THF under N_2_ or CO_2_ (Fig. 2a) exhibit absorption band at 298 nm, attributable to the ππ* transition of opda and a transition with ππ* character of **1**^51^. In the case of opda, spectral differences were not observed under N_2_ or CO_2_, indicating a negligible effect of the atmosphere on the electronic state. Even though the molar absorption coefficient of **1** changed slightly from N_2_ to CO_2_ (Fig. 2a), the similarity of the spectra and the fact that the same colorless crystals of **1** were obtained from THF/*n*-hexane under each atmosphere (Supplementary Fig. 1 and Supplementary Table 1) indicate that **1** is stable under both gases. When the solution was left to stand for 8 h under CO_2_, there were virtually no UV-Vis spectral differences in both opda and **1** (Supplementary Fig. 2), demonstrating their stability in the dark. The ^1^H NMR (THF-*d*_8_) spectra of opda under N_2_ or CO_2_ (Supplementary Fig. 3) show characteristic signals at 6.42, 6.50, and 3.80 ppm, assignable to aromatic C–H and N–H protons. On the other hand, the spectra of **1** under N_2_ or CO_2_ (Supplementary Fig. 3) did not show any clear signals, reflecting its paramagnetic nature^51^. Aromatic amines afford carbamic acids or benzimidazol-2-one from the reaction with CO_2_ under basic conditions^52,53^ or in the presence of catalysts^54–56^. However, such products could not be detected under these dark conditions.

When a THF solution of opda was irradiated with UV light (*λ*_ex_ = 300 ± 10 nm) under N_2_, PHE was observed with an apparent quantum yield of *Φ*_H2@N2_(%) = 1.28 × 10^−3^ (8 h) (Fig. 2b). Contrastively, the amount of evolved hydrogen under CO_2_ decreased by one third under N_2_ (*Φ*_H2@CO2_(%) = 4.16 × 10^−4^) (8 h). Under these conditions, a THF solution of **1** also showed PHE with *Φ*_H2@N2_(%) = 0.0138 (8 h) and *Φ*_H2@CO2_(%) = 3.56 × 10^−3^ (8 h) (Fig. 2b). The observed inhibitions of the PHE under CO_2_ clearly suggest an alternative photochemical process under CO_2_ compared to that under N_2_.

### Direct photochemical carboxylation of aromatic diamines with CO_2_ by opda and 1

**Figure 3:**
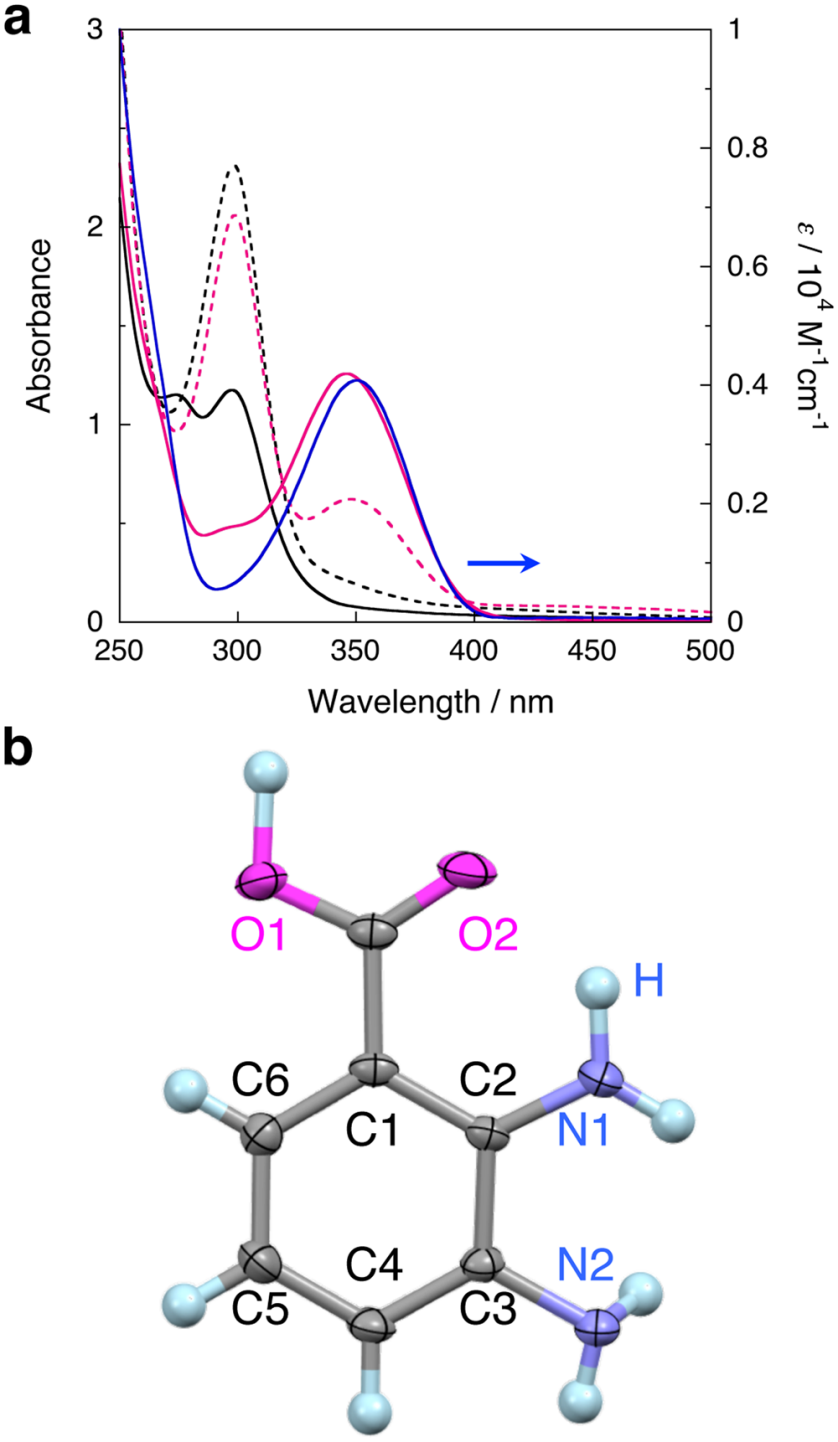
(**a**)UV-Vis spectra of opda (6.0 × 10^−3^ M) after irradiation (*λ*_ex_ = 300 ± 10 nm; 63.9–66.9 mW) under CO_2_ () or N_2_ (···), and those of **1** (2.0 × 10^−3^ M) under CO_2_ () or N_2_ (—) in THF (8 h; room temperature), together with spectrum of commercial DBA (). (**b**) Crystal structure of DBA (unit A); atomic displacement parameters set to 50% probability; color-code: blue = N, gray = C, magenta = O, and light blue = H; hydrogen atoms are depicted in ball-and-stick mode for clarity.

The UV-Vis spectra of opda and **1** after photo-irradiation (8 h) under CO_2_ showed new absorption band at 347 nm (Fig. 3a). It is noteworthy that the new band was observed only when the photoreaction took place under CO_2_ (Supplementary Fig. 2). Interestingly, the absorbance of the new bands was significantly increased for **1** relative to opda, which confirms the promoting effect of the Fe(II) ion in **1**. The ^1^H NMR (CD_3_CN) spectrum after the photoreaction of opda under CO_2_ (Supplementary Fig. 4) exhibits two sets of doublets at 6.82 and 7.30 ppm, as well as a triplet at 6.51 ppm, suggesting the formation of a 1,2,3-trisubstituted benzene. A similar spectrum was observed after the photoreaction of **1** under identical conditions (Supplementary Fig. 4). X-ray diffraction analysis of the pale yellow crystals of the product unambiguously identified 2,3-diaminobenzoic acid (DBA) (Fig. 3b and Supplementary Fig. 5, Supplementary Tables 2 and 3)^57,58^. Interestingly, one *ortho* C–H bond relative to an amino group was carboxylated. The similarity between the UV-Vis and ^1^H NMR spectra of commercial DBA and the photochemical products of opda or **1** (Fig. 3a and Supplementary Fig. 6) indicates that DBA is the main product, while the isomer, i.e., 3,4-diaminobenzoic acid, was not obtained in detectable amounts. Based on the molar absorption coefficient of DBA (*ε* = 4,041 M^−1^ cm^−1^) at 347 nm (Fig. 3a), the photochemical reaction of **1**, prepared *in-situ* by mixing [Fe^II^(H_2_O)_6_][ClO_4_]_2_ and 3 eq. of opda (run 3 in Table 1), afforded DBA in 58.0% (*Φ*(%) = 0.47), which is comparable to that for *ex-situ-*prepared **1** (54.1%; *Φ*(%) = 0.44**)** (Supplementary Fig. 7 and run 1 in Table 1). On the other hand, the photo-irradiation of a THF solution of opda afforded DBA in 27.5% (*Φ*(%) = 0.22) (Fig. 3a and run 4 in Table 1).

**Table 1:** Results of the photo-irradiation of aromatic amines under CO_2_^a^.

Run	Substrate	Metal	*λ*_max_ (nm)	*λ*_*ex*_ (nm)	Product	Yield(%)^b^	*Φ*(%)^c^
1^d^		Fe(II)	298	300 ± 10		54.1	0.44
2^d^		Fe(II)	298	—	N.D.^e^	—^g^	—^g^
3		Fe(II)	298	300 ± 10		58.0	0.47
4		—	298	300 ± 10		27.5	0.22
5		Fe(II)	291	289 ± 10	N.D.^e^	—^*g*^	—^g^
6		—	298	300 ± 10		trace	—^g^
7		Fe(II)	—^f^	— ^f^	— ^f^	— ^f^	—^f^
8		—	324	300 ± 10		trace	—^g^
9		Fe(II)	—^f^	—^f^	—^f^	—^f^	—^f^

^a^Reaction time: 8 h; light power: 63.5–66.9 mW (*λ*_ex_ = 300 ± 10 nm) and 55.1 mW (289 ± 10 nm). ^b^Estimated based on the amounts of used aromatic amines and absorbance of the products. ^c^Apparent quantum yield (%). ^d^The *ex-situ*-prepared Fe complex was used. ^*e*^Not detected. ^f^The reaction was not investigated due to the formation of precipitates after mixing [Fe^II^(H_2_O)_6_][ClO_4_]_2_ and 3 eq. of the substrates. ^g^Not estimated.

### A reaction mechanism for the photochemical carboxylation

To get insight into the underlying reaction mechanisms, we subsequently carried out the photoreaction with aniline and Fe(II) under CO_2_. However, the UV-Vis spectrum showed no significant changes (Supplementary Fig. 8 and run 5 in Table 1). The inertness of aniline prompted us to use *m-* (mpda) and *p*-phenylenediamine (ppda)^59,60^. The photo-irradiations (*λ*_ex_ = 300 ± 10 nm) of mpda or ppda resulted in the emergence of new absorbances at 360 nm and 400 and 450 nm, respectively (Supplementary Fig. 9, runs 6 and 8 in Table 1). Curiously, the absorbances of mpda and ppda were observed even after irradiation, suggesting their poor reactivity (Supplementary Fig. 9). ESI-MS spectra showed signals (*m*/*z* 151.05) for the carboxylated products in the crude reaction mixture (Supplementary Figs 10a and 11a). The newly emerged ^1^H NMR signals of the carboxylated products were assigned to 2,4- and 2,5-diaminobenzoic acids (Supplementary Figs 10b and 11b). Conversely, the treatment of mpda or ppda with Fe(II) afforded white precipitates, probably due to the formation of coordination polymers (runs 7 and 9 in Table 1)^61,62^.

Given the atmosphere-dependent photoreactions of opda and **1**, we focused our attention on their excited states. The emission spectra (*λ*_ex_ = 300 nm) of opda and **1** in THF under N_2_ or CO_2_ showed the emission bands at 350 nm, assignable to emissions from ππ* of opda or ππ* included excited state of **1** (Supplementary Fig. 12). In the excitation spectra of opda and **1** under N_2_ or CO_2_ (*λ*_obs_ = 350 nm), the bands were observed at 298 nm, suggesting radiative deactivation pathways for the photoreactions under N_2_ and CO_2_ (Supplementary Fig. 13).

**Figure 4:**
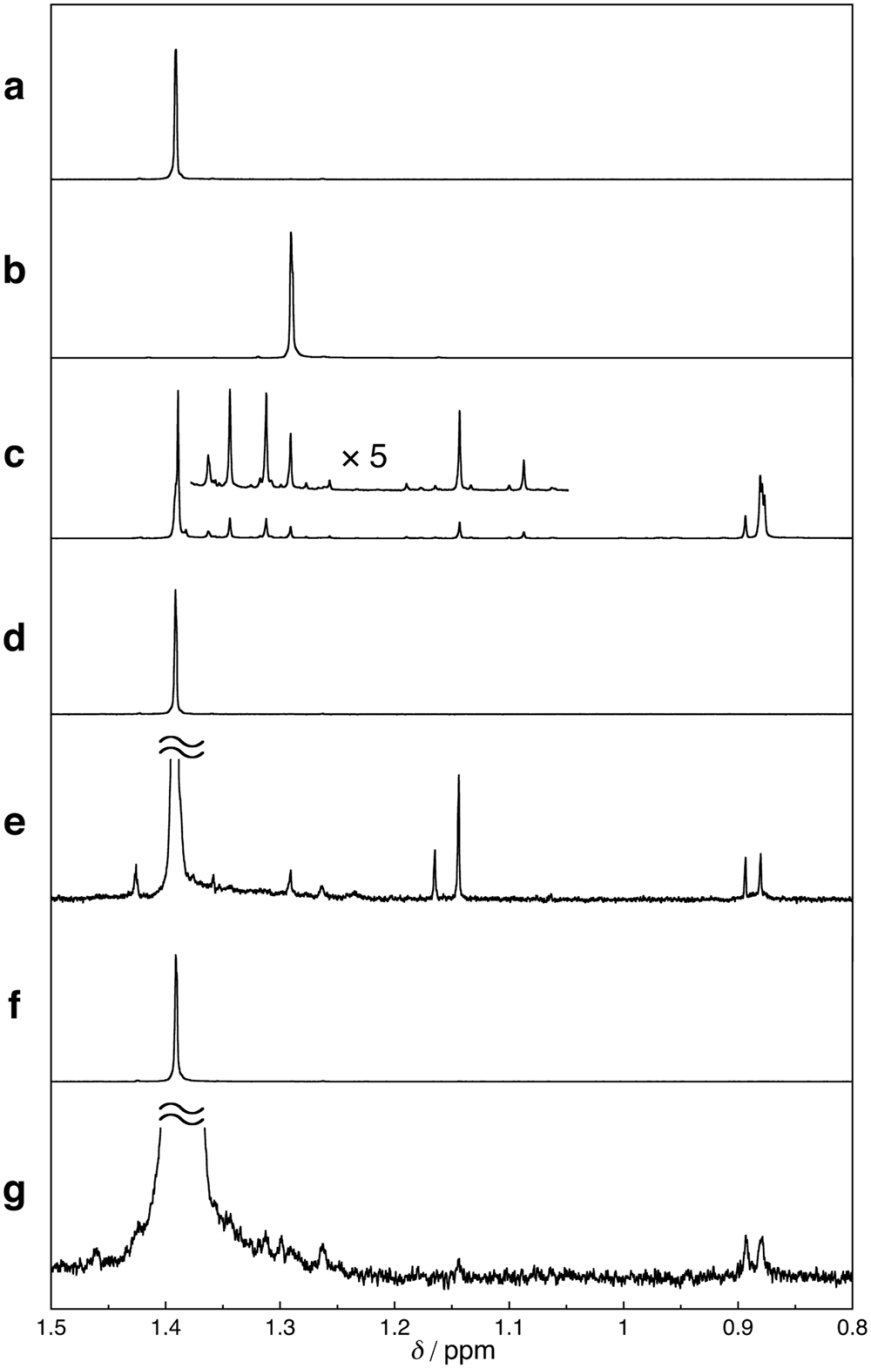
^1^H NMR (THF-*d*_8_, 500 MHz) spectra of (**a**) *t*-BuSH, (**b**) *t*-Bu_2_S_2_, (**c**) *t*-Bu_2_S_2_ after photo-irradiation (*λ*_ex_ = 300 ± 10 nm; 64.5 mW; 3 h), (**d**) *t*-BuSH with opda, (**e**) *t*-BuSH after photoirradiation (*λ*_ex_ = 300 ± 10 nm; 64.2 mW; 3 h) in the presence of opda, (**f**) *t*-BuSH with **1**, and (**g**) *t*-BuSH after photo-irradiation (*λ*_ex_ = 300 ± 10 nm; 64.5 mW; 3 h) in the presence of **1**, in THF-*d*_8_ under CO_2_.

Subsequently, we attempted to identify the active species by trapping experiments. It was previously reported that 2-methylpropane-2-thiol (*t*-BuSH) can act as a hydrogen (H) radical scavenger forming di-*tert*-butyl disulfide (*t*-Bu_2_S_2_)^63,64^. The detection of *t*-Bu_2_S_2_ among the photochemical reaction products of opda and **1** revealed the H radical generation during the reaction (*vide infra*). The ^1^H NMR spectra of *t*-BuSH and *t*-Bu_2_S_2_ under CO_2_ (Fig. 4a,b, and Supplementary Fig. 14) showed singlets at 1.38 and 1.29 ppm, respectively. On the other hand, we found that the new signals emerged at 0.88, 0.89, and 1.19 ppm in the ^1^H NMR spectrum of a THF-*d*_8_ solution of *t*-Bu_2_S_2_ after photo-irradiation (*λ*_ex_ = 300 ± 10 nm), which demonstrates the photoreactivity of *t*-Bu_2_S_2_ (Fig. 4c)^63^. These resonances are thus indicative of the *in-situ* formation of *t*-Bu_2_S_2_. A mixture of opda/*t*-BuSH displayed a ^1^H NMR spectrum similar to those of pure *t*-BuSH and opda (Fig. 4d and Supplementary Fig. 14), suggesting negligible interactions in the ground state. After photo-irradiation, new singlets emerged at 0.88, 0.89, and 1.19 ppm (Fig. 4e), and these peaks are identical to those of the photochemical products derived from *t*-Bu_2_S_2_ (Fig. 4c), suggesting the formation of *t*-Bu_2_S_2_ during the photochemical reaction. The ^1^H NMR spectrum of a mixture of **1**/*t*-BuSH showed no significant interaction in the ground state (Fig. 4f), whereas new singlets emerged at 0.88 and 4.61 ppm after photo-irradiation (Fig. 4g and Supplementary Fig. 14). These peaks are comparable to those of the photochemical products of opda/*t*-BuSH mixture (Fig. 4e), suggesting the formation of *t*-Bu_2_S_2_ from **1**/*t*-BuSH. Based on these results, it should be feasible to consider a reaction pathway involving the H radical generation for the photoreaction of opda and **1** under CO_2_. The lower amount of photochemically generated H_2_ from them under CO_2_ than N_2_ thus most likely reflects the incorporation of the generated H radicals in the DBA skeleton.

**Figure 5:**
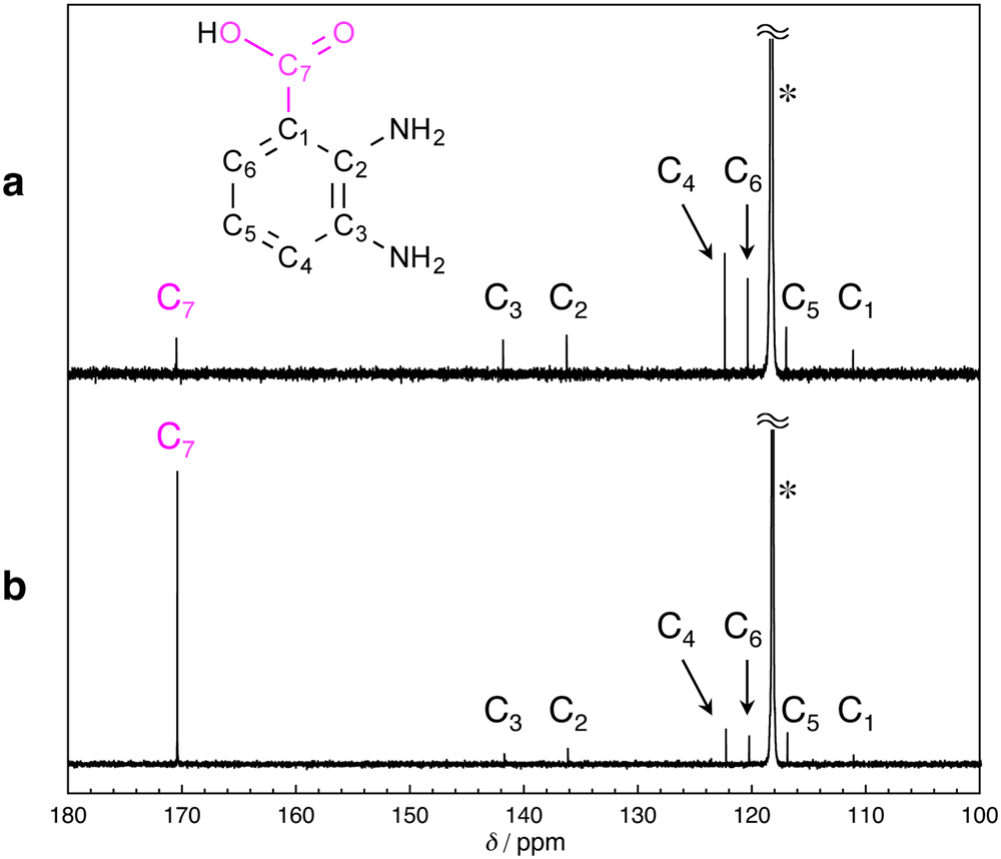
^13^C NMR (CD_3_CN, 126 MHz) spectra of the photochemical product (*λ*_ex_ = 300 ± 10 nm) of **1** under (**a**) CO_2_ and (**b**) ^13^CO_2_ in THF at room temperature. The symbol “*” indicates the ^13^C NMR signals of CD_3_CN.

Finally, to shed more light on the reaction mechanism, we compared the ^13^C NMR (CD_3_CN) spectra of the reaction product of **1** under CO_2_ or ^13^CO_2_. In the ^13^C NMR spectrum of the photochemically-produced DBA from **1** under CO_2_, the resonance derived from the carboxyl carbon was observed at 170.5 ppm (Fig. 5a). In the case of the photoreaction under ^13^CO_2_, the peak intensity of the carboxyl carbon clearly increased, suggesting that the carboxyl moiety in DBA originates from CO_2_ (Fig. 5b)^14,65^.

**Figure 6:**
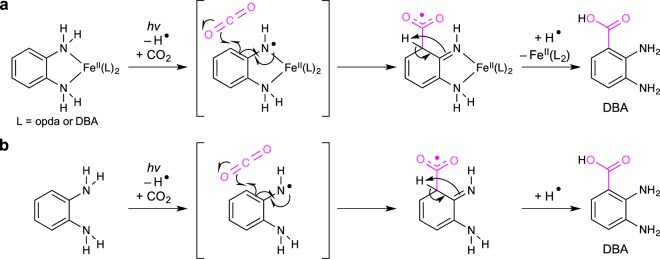
Plausible reaction mechanisms for the photo-induced direct carboxylation of opda in the (**a**) presence and (**b**) absence of Fe(II).

Figure 6 depicts plausible mechanisms for the photochemical carboxylation of opda and **1**. Given the aforementioned results, the photo-irradiation induces the generations of H and aminyl radical intermediates. The later then form a C–C bond with CO_2_ *via* the delocalization of the unpaired electron, thus forming the carboxyl radical intermediate^66^. Subsequently, the methine proton transferrs to the imino nitrogen, whereby the aromatic stabilization could act as driving force forming a 2,3-diaminobenzoic radical species. The reaction of the intermediate with a H radical might finally yield DBA. The role of the Fe(II) ion in this reaction should be worth investigating in detail, as it is highly plausible that the Fe(II) center perturbs the N–H moiety in opda favorably^64,67–71^.

## Discussion

In this paper, we demonstrated a direct photochemical C–H carboxylation of aromatic diamines with CO_2_. Although this reaction is not catalytic, it represents the first example of atom- and step-economic direct carboxylation of a C–H bond in benzene rings in the absence of any potentially reactive electron donor and base. The promotion of this reaction by Fe(II) could be achieved using opda ligand, indicating the potential of nonprecious metal ions to accelerate or catalyze the reaction. Further efforts to gain an in-depth understanding of the mechanism and to expand the scope of the reaction by using a wider range of aromatic polyamines, nonprecious metal ions, excitation wavelengths, and catalytic protocols are currently in progress.

## Methods

### General procedures

All synthetic operations were performed under N_2_ or CO_2_ using standard Schlenk-line techniques. The ligand opda was purchased from Wako Pure Chemical Industries (Japan), while *p*-phenylenediamine (ppda) was obtained from Sigma-Aldrich, and *m*-phenylenediamine (mpda), was procured from Tokyo Chemical Industry Co., Ltd. (Japan). 2,3-Diaminobenzoic acid (DBA) was purchased from Combi-blocks (USA) and used after recrystallization from H_2_O. Dehydrated THF, THF-*d*_8_, CD_3_CN, and silica gel (60 N) were purchased from Kanto Chemical Co. Inc. (Japan). MeOH, CH_3_CN, and emission analysis grade THF were obtained from Nacalai Tesque, Inc. (Japan). N_2_, CO_2_, and ^13^CO_2_ were purchased from Kotobuki Sangyo Co. Ltd. (Japan). Prior to use, THF was degassed by at least five freeze-pump-thaw cycles, followed by N_2_ or CO_2_ sparging for 20 min, and subsequent dehydration over molecular sieves (4 Å, MS4A), which were purchased from Wako Pure Chemical Industries (Japan) and activated by heating under high vacuum. Complex [Fe^II^(opda)_3_][ClO_4_]_2_ (**1**) was prepared according to a previously reported procedure^51^. Caution! Although we did not experience any difficulties manipulating perchlorate salts, these should be regarded as potentially explosive and therefore require handled with the utmost care.

UV-Vis-NIR spectra were recorded on a HITACHI U-4100 spectrophotometer at room temperature (25 °C). IR spectra were recorded on a Thermo Nicolet 6700 FT-IR spectrometer by attenuated total reflection (ATR) method. ^1^H and ^13^C NMR (500 and 126 MHz) spectra were recorded on a JEOL EX-500 (and A-500) spectrometer using CD_3_CN or THF-*d*_8_. Elemental analyses were carried out on a Perkin-Elmer 2400 II CHN analyzer. Electrospray ionization mass spectra (ESI-MS) were performed at the Global Facility Center at Hokkaido University. Emission and excitation spectra were recorded on a Horiba FluoroMax-4 spectrophotometer at room temperature (25 °C).

### Preparation of the solutions used for the reactions under atmospheres of N_2_ or CO_2_ (in the dark or under irradiation)

The sample solutions for reactions under N_2_ or CO_2_ were prepared under the respective atmospheres. A colorless THF solution (2.0 mM) of *ex-situ-*prepared [Fe^II^(opda)_3_][ClO_4_]_2_ was obtained from dissolving [Fe^II^(opda)_3_][ClO_4_]_2_ (6.51 mg, 1.0 × 10^−5^ mol) in THF (5 mL). On the other hand, *in-situ*-prepared [Fe^II^(opda)_3_][ClO_4_]_2_ was obtained from the treatment of [Fe^II^(H_2_O)_6_][ClO_4_]_2_ (3.63 mg, 1.0 × 10^−5^ mol) with opda (3.24 mg, 3.0 × 10^−5^ mol) in THF (5 mL). In a similar manner, a colorless THF solution of a mixture of [Fe^II^(H_2_O)_6_][ClO_4_]_2_ and aniline was prepared using aniline (2.73 µL, 3.0 × 10^−3^ mol) instead of opda. THF solutions (6 mM) of Fe^II^-free opda, aniline, mpda, or ppda were prepared by dissolving opda (3.24 mg, 3.0 × 10^−5^ mol), aniline (2.73 µL, 3.0 × 10^−3^ mol), mpda (3.24 mg, 3.0 × 10^−5^ mol), or ppda (3.24 mg, 3.0 × 10^−5^ mol) in THF (5 mL).

To investigate the reactivity under photo-irradiation, 0.4 mL of the respective sample solutions were transferred into a N_2_- or CO_2_-filled 1 mm quartz cell and the UV-Vis spectrum of the initial state was measured. The remaining 4 mL of the sample solution were then transferred into a custom-made Schlenk-flask-equipped quartz tube (volume: 164 mL). After exposing this apparatus for 8 h to photo-irradiation, 0.4 mL of the sample solution were withdrawn and transferred into a N_2_- or CO_2_-filled 1 mm quartz cell in order to measure the UV-Vis spectrum. In order to examine the reactivity in the dark, 0.4 mL of the solution were transferred into a N_2_- or CO_2_-filled 1 mm quartz cell and the UV-Vis spectrum of the initial state was measured. After allowing the sample solutions to stand for 8 h, in the dark, the spectral measurements were recorded again.

### Photochemical hydrogen evolution

For the photochemical hydrogen-evolution reaction (HER), the aforementioned Schlenk-flask-equipped quartz tube (volume: 164 mL) and THF solutions (4 mL) were used. The light source for the photochemical reactions was a 200 W Hg-Xe lamp (LC-8, Hamamatsu Photonics K.K.), and the intensity of the light was measured by using a power meter (Nova, Ophir Optronics Ltd.) and a thermopile sensor (3 A, Ophir Optronics Ltd.) prior to photo-irradiation experiments. Gas chromatographic analyses were conducted using a Shimadzu gas chromatograph (GC-2014) equipped with a thermal conductivity detector (TCD), a column filled with 5 Å molecular sieves, and Ar as the carrier gas (15.0 mL/min). The oven temperature was maintained at 100 °C, while the column and detector temperatures were set to 70 °C and 200 °C, respectively. Before the photo-irradiation experiments, a gas sample (0.3 mL) was collected from the headspace using a gas-tight syringe (Tokyo Garasu Kikai Co. Ltd) and analyzed by GC to confirm the successful N_2_ or CO_2_ purge. The samples were then exposed to irradiation in a water bath at room temperature. During the reaction, gas samples (0.3 mL) were collected from the headspace in order to determine the amount of H_2_ evolved as a function of the irradiation time.

### Purification of DBA after the photoreactions

After the reactions in the dark or upon photo-irradiation, as well as measurements of UV-Vis spectra of the samples after the reaction, all THF solutions were transferred into a Schlenk flask and THF was removed under reduced pressure. After measuring of the ^1^H NMR and ESI-MS spectra, the reaction mixtures were purified by flash column chromatography (Isolera One ACI^TM^ Spektra, Biotage Co. Ltd.) on silica gel (60 N; Kanto Chemical Co. Inc.; eluent: CH_3_CN:MeOH = 9:1 then 0:10). The photochemical products of [Fe^II^(opda)_3_][ClO_4_]_2_ or Fe^II^-free opda were collected and dried *in vacuo*. The formation of DBA was confirmed by recording the ^1^H NMR spectra in CD_3_CN. Colorless single crystals of DBA suitable an X-ray crystallographic analysis were obtained from a recrystallization from THF/*n*-hexane.

### Calculation of the quantum yields (*Φ*%)

The THF solutions of the samples, except for those of aniline and a mixture of aniline and [Fe^II^(H_2_O)_6_][ClO_4_]_2_, were exposed to photo-irradiation (*λ*_ex_ = 300 ± 10 nm) from a Hg-Xe lamp equipped with the LX0300 band pass filter (Asahi Spectra Inc.; *λ* = 300 ± 10 nm; half bandwidth = 10.40 nm). The THF solutions of aniline and a mixture of aniline and [Fe^II^(H_2_O)_6_][ClO_4_]_2_ were exposed to photo-irradiated (*λ*_ex_ = 289 ± 10 nm) from a Hg-Xe lamp equipped with a CWL289 nm filter (OptoSigma Corporation, *λ* = 289 ± 10 nm, half bandwidth = 10 nm). The amount of DBA formed in runs 1, 3, and 4 (Table 1) in the subsequent 8 h were used to calculate the apparent quantum yield (*Φ*) using Eq. 1.

1 $${\Phi }={N}_{{\rm{e}}}/{N}_{{\rm{p}}}={N}_{{\rm{DBA}}}/{N}_{{\rm{p}}}$$

where, *N*_e_ refers to the number of reacted electrons, *N*_DBA_ to the number of molecules of DBA formed in the reaction, and *N*_p_ to the number of incident photons.

## Electronic supplementary material

Supplementary Information

Supplementary Dataset 1

Supplementary Dataset 2

## Data Availability

The X-ray crystallographic coordinates for the structure of DBA reported in this Article has been deposited at the Cambridge Crystallographic Data Centre (CCDC), under deposition number CCDC-1826028. These data can be obtained free of charge from The Cambridge Crystallographic Data Centre *via* http://www.ccdc.cam.ac.uk/data_request/cif.
