# Supplementary material for: Direct Photochemical C–H Carboxylation of Aromatic Diamines with CO2 under Electron-Donor- and Base-free Conditions
Source: Sci Rep. 2018 Oct 2;8:14623. doi: 10.1038/s41598-018-33060-3 (PMC6168591; doi:10.1038/s41598-018-33060-3)
Supplement: Supplementary file 1 — Supplementary Information [file 41598_2018_33060_MOESM1_ESM.pdf]

Supplementary Information

for

Direct Photochemical C–H Carboxylation of Aromatic Diamines with CO_2 under Electron-Donor- and Base-free Conditions

Takeshi Matsumoto,[1,2] Daiki Uchijo,[1] Takuji Koike,[1] Ryoya Namiki,[1] Ho-Chol Chang[1] *

[1] Department of Applied Chemistry, Faculty of Science and Engineering, Chuo University, 1-13-27 Kasuga, Bunkyo-ku, Tokyo 112-8551, Japan

[2] Precursory Research for Embryonic Science and Technology (PRESTO), Japan Science and Technology Agency (JST), 4-1-8 Honcho, Kawaguchi, Saitama 332-0012, Japan

Correspondence and requests for materials should be addressed to H.C. E-mail: chang@kc.chuo-u.ac.jp

Phone: +81-3-3817-1897. Fax: +81-3-3817-1895.

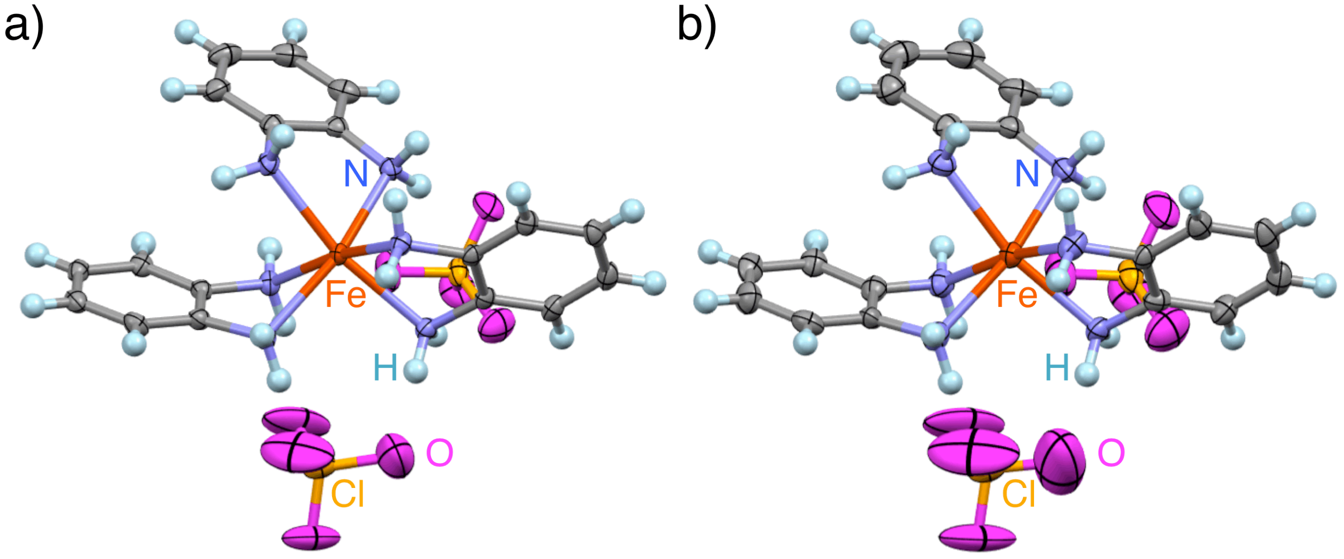

Supplementary Fig. 1 Crystal structures of **1** obtained from under a) N_2[1] or b) CO_2. Atomic displacement parameters set to 50% probability; color code: brown = Fe, blue = N, gray = C, magenta = O, orange = Cl, and light blue = H; hydrogen atoms are depicted in ball-and-stick mode, while one molecule of THF included in the crystals is omitted for clarity.

An X-ray crystallographic analyses of the colorless crystals of **1** obtained from THF/*n*-hexane under CO_2 was conducted to confirm the influence of the CO_2 atmosphere on the structure of **1**. Previously, the structure of **1** has been identified as $[Fe^{II}(opda)_3][ClO_4]_2$·THF,[1] in which three opda ligands coordinate to the *high-spin* (*hs*) Fe^{II} center using a bidentate coordination mode, using single crystals that were obtained from a THF/*n*-hexane solution of **1** under N_2 (Supplementary Fig. 1a). We found that the cell parameters at 183 K obtained from crystals grown under CO_2 were almost identical to those of $[Fe^{II}(opda)_3][ClO_4]_2$·THF grown under N_2 (Supplementary Table 1). In fact, the determined structure shown in Supplementary Fig. 1b is similar to that of $[Fe^{II}(opda)_3][ClO_4]_2$·THF, suggesting that the structure of $[Fe^{II}(opda)_3][ClO_4]_2$ is maintained in THF, regardless of the presence of N_2 or CO_2.

Supplementary Table 1 Crystallographic data for 1 using single crystals obtained from THF/hexane under either N_2 or CO_2.

Atmosphere	N_2[a]	CO_2
Formula	$C_{22}H_{32}Cl_2FeN_6O_9$	$C_{22}H_{32}Cl_2FeN_6O_9$
FW	651.28	651.28
Crystal size (mm^3)	0.30×0.16×0.16	0.20×0.20×0.20
Crystal system	monoclinic	monoclinic
Space group	$P2_1/n$ (No. 14)	$P2_1/n$ (No. 14)
a (Å)	17.849(2)	17.9219(14)
b (Å)	8.3097(9)	8.3603(7)
c (Å)	18.998(2)	19.0215(15)
α (°)	90	90
β (°)	100.5257(15)	100.846(8)
γ (°)	90	90
V (Å^3)	2770.4(6)	2799.1(4)
T (K)	150	183
Z	4	4
D_{calc} (g cm^{-3})	1.561	1.545
F (000)	1352	1352
μ(Mo Kα) (cm^{-1})	7.974	7.892
Measured reflections	22454	25501
Unique reflections	6242	8355
Refined parameters	362	361
GOF on F^2	1.125	1.009
R_{int}	0.048	0.1274
R_1[b]	0.0566	0.0956
wR_2[c] (all data)	0.1533	0.3085

[a] Ref. 1. [b] $R_1 = \Sigma||F_o| - |F_c||/\Sigma|F_o|$. [c] $wR_2 = \{[\Sigma w(F_o^2 - F_c^2)^2]/[\Sigma w(F_o^2)^2]\}^{1/2}$.

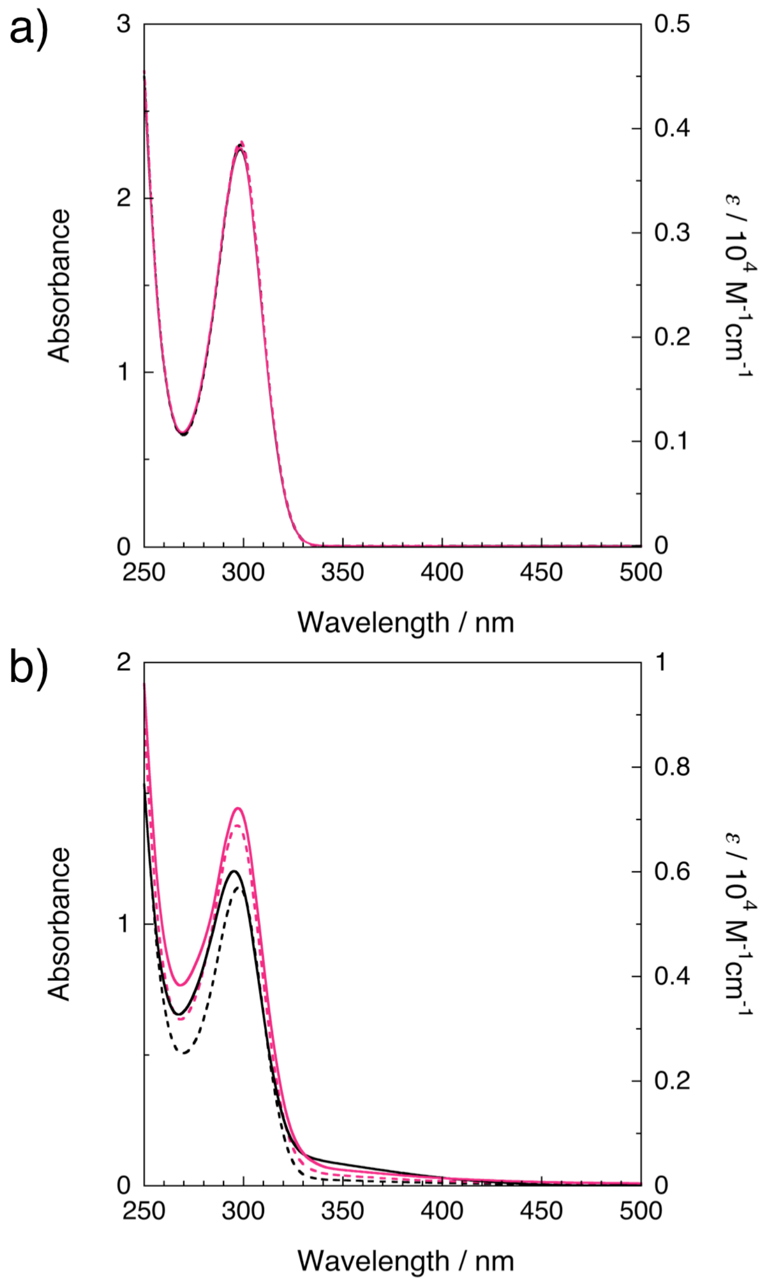

Supplementary Fig. 2 Absorption spectra for a) opda (6.0×10^{-3} M) and b) **1** (2.0×10^{-3} M) in THF under CO_2 (···) or N_2 (···), as well as those after allowing the sample solutions to stand for 8 h under CO_2 (–) or N_2 (–) in the dark.

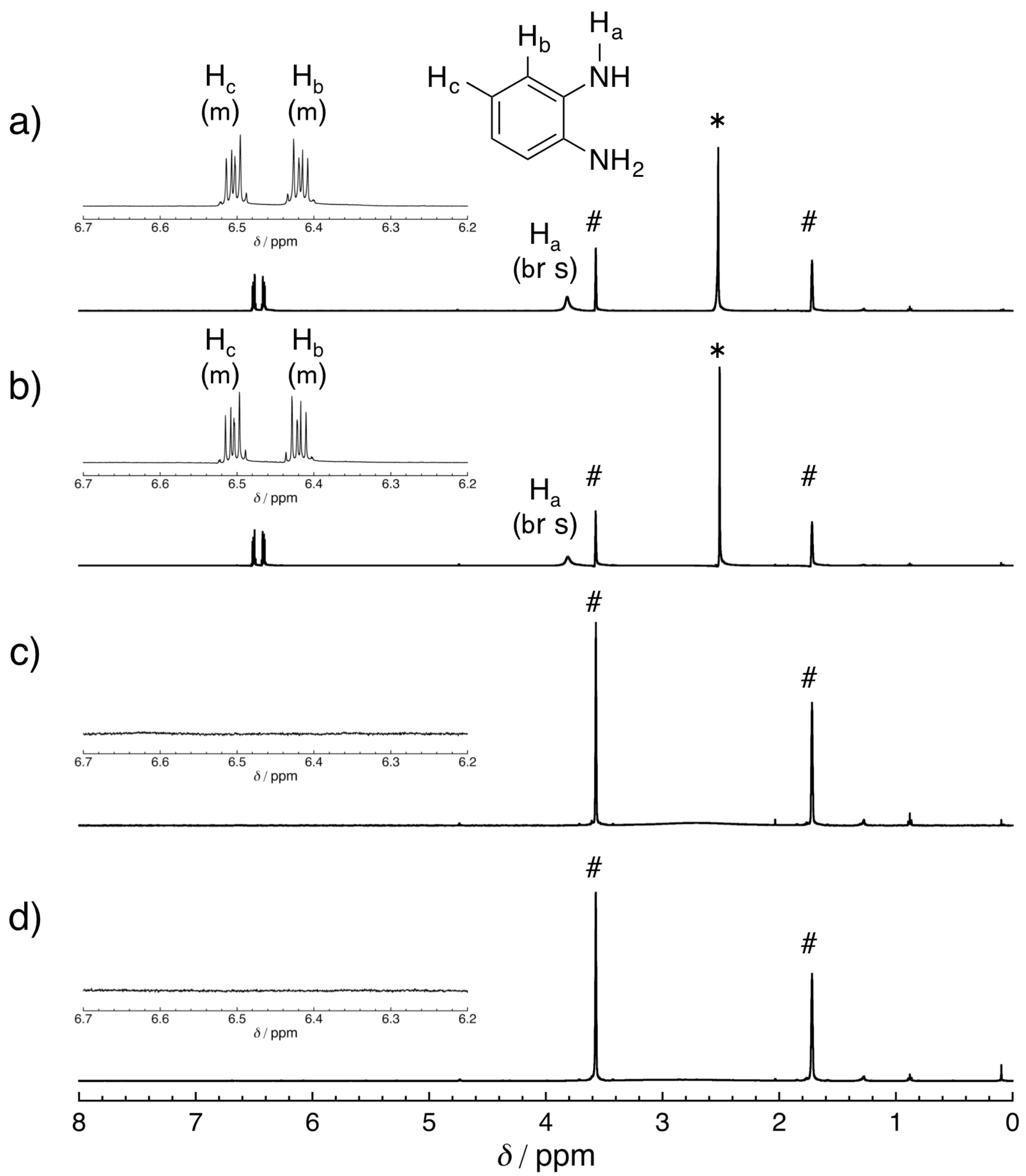

Supplementary Fig. 3 ^{1}H NMR (THF-d_8, 500 MHz) spectra of opda under a) N_2 or b) CO_2, as well as **1** under c) N_2 or d) CO_2. The insets show the magnified spectral region of 6.2–6.7 ppm. The symbols "#" and "*" indicate the signals from residual THF-d_8 and H_2O, respectively.

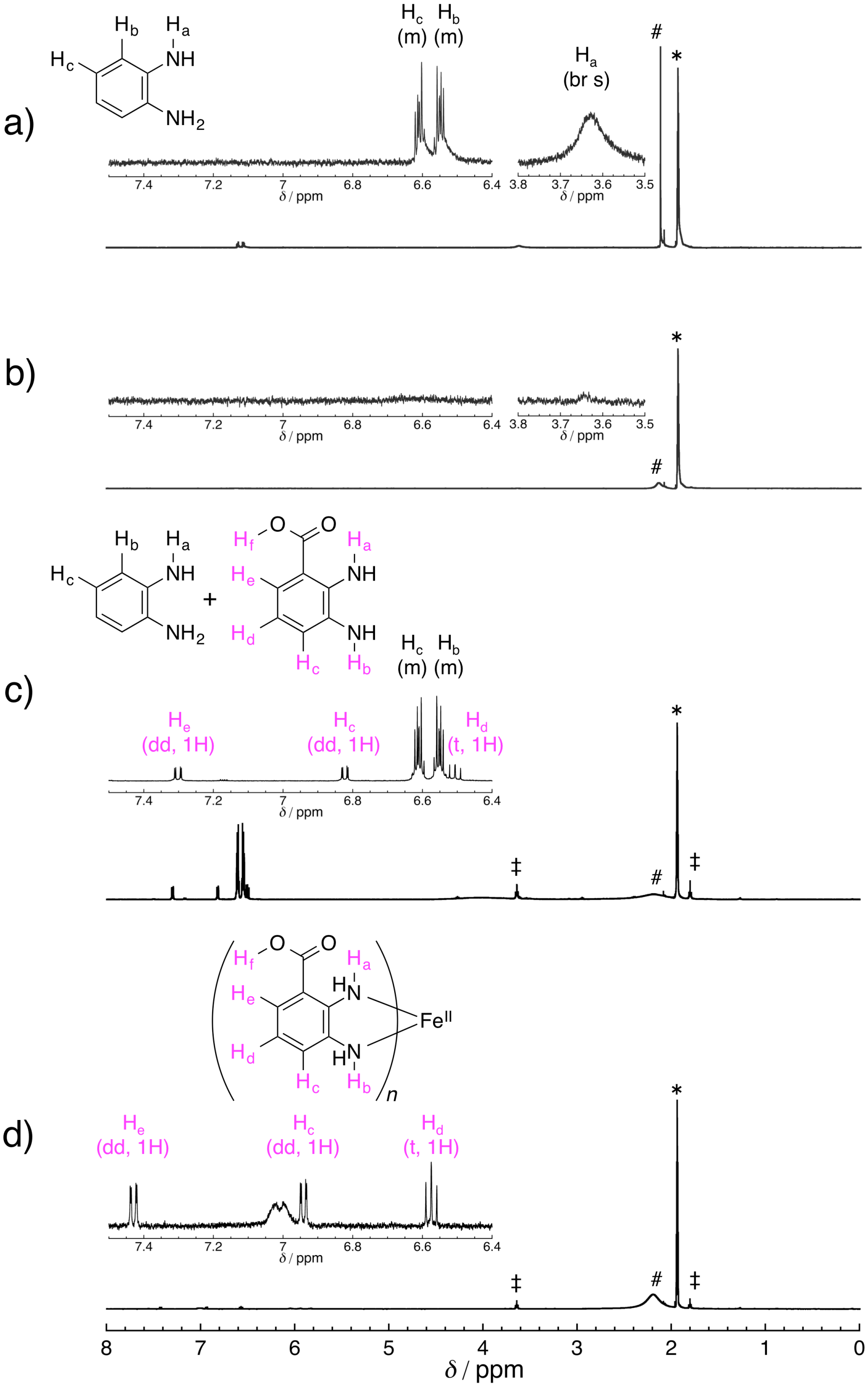

Supplementary Fig. 4 ^{1}H NMR (CD_3CN, 500 MHz) spectra of a) opda and b) **1**, as well as of the photochemical products of c) opda (6.0×10^{-3} M, 8 h), and d) **1** (2.0×10^{-3} M, 8 h) at room temperature under CO_2. The insets show the magnified spectral region of 6.4–7.5 ppm. The symbols "*", "#", and "‡" indicate signals from residual protons in CD_3CN, H_2O, and THF, respectively. Amino and carboxylic protons of opda and DBA in c) and d) could not be detected, which is probably due to the hydrogen-bonding interactions with residual H_2O molecules.

Supplementary Table 2 Crystallographic data for DBA.

Formula	$C_7H_8N_2O_2$
FW	152.15
Crystal size (mm^3)	0.17×0.06×0.04
Crystal system	monoclinic
Space group	$P2_1/c$ (No. 14)
a (Å)	8.646(5)
b (Å)	11.344(6)
c (Å)	13.893(8)
α (°)	90
β (°)	99.209(10)
γ (°)	90
V (Å^3)	1345.1(13)
T (K)	93
Z	8
D_{calc} (g cm^{-3})	1.503
F (000)	640.00
μ(Mo Kα) (cm^{-1})	1.127
Measured reflections	10759
Unique reflections	3045
Refined parameters	239
GOF on F^2	1.013
R_{int}	0.0481
R_1 [a]	0.0627
wR_2 [b] (all data)	0.1790

[a] $R_1 = \Sigma||F_o| - |F_c||/\Sigma|F_o|$.

[b] $wR_2 = \{[\Sigma w(F_o^2 - F_c^2)^2]/[\Sigma w(F_o^2)^2]\}^{1/2}$.

Supplementary Table 3 Selected bond distances and angles in DBA.

Bond distances (Å)			
**Unit A**		**Unit B**	
N1–C2	1.371(3)	N3–C9	1.367(3)
N2–C3	1.432(3)	N4–C10	1.457(3)
C1–C2	1.413(3)	C8–C9	1.424(3)
C1–C6	1.406(3)	C8–C13	1.394(3)
C1–C7	1.480(3)	C8–C14	1.500(3)
C2–C3	1.418(3)	C9–C10	1.412(3)
C3–C4	1.373(3)	C10–C11	1.379(3)
C4–C5	1.394(3)	C11–C12	1.382(3)
C5–C6	1.373(3)	C12–C13	1.388(3)
O1–C7	1.326(3)	O3–C14	1.284(3)
O2–C7	1.233(3)	O4–C14	1.248(3)
Angles (°)			
O1–C7–O2	122.84(18)	O3–C14–O4	123.13(17)

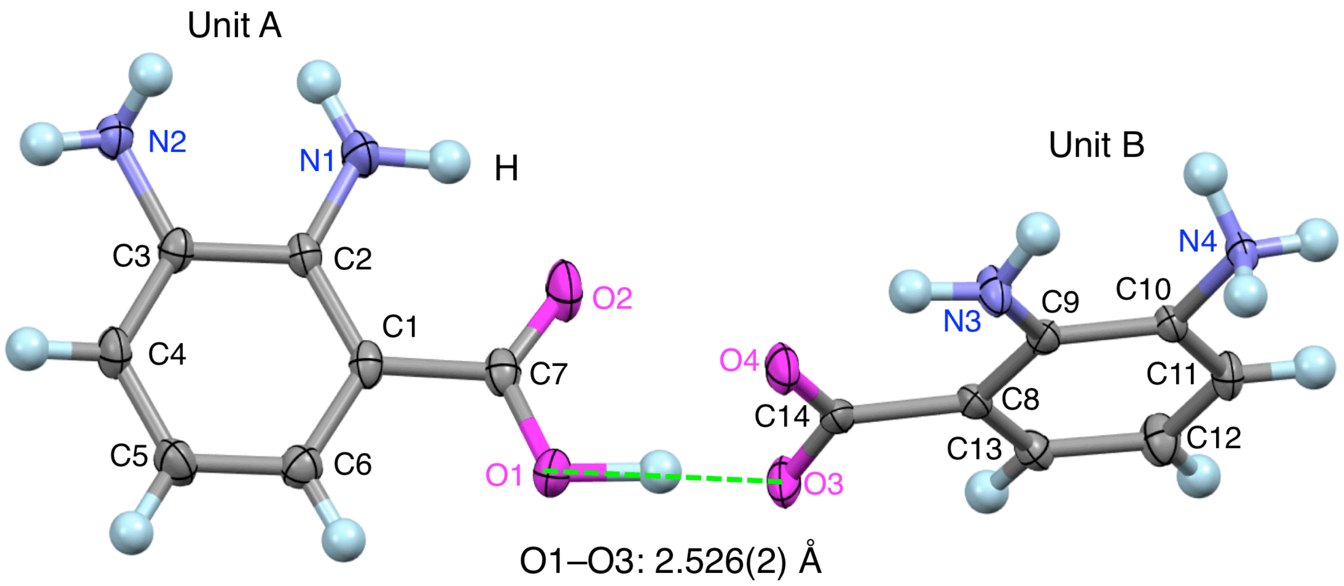

Supplementary Fig. 5 Crystallographically independent units A and B in the unit cell of DBA. Atomic displacement parameters set at 50% probability; color code: blue = N, gray = C, red = O, and light blue = H; hydrogen atoms are depicted in the ball-and-stick mode.

Subjecting the photochemically generated crude product of **1** to flash column chromatography and recrystallization from THF/*n*-hexane afforded pale-yellow crystals of DBA, which contained two crystallographically independent, yet structurally similar units A and B in the unit cell. The N1–C2 and N3–C9 bonds were close to typical N–C single bonds (1.372–1.381 Å; Supplementary Table 3).[2,3] On the other hand, the N2–C3 and N4–C10 bonds were slightly longer than those of N1–C2 and N3–C9 due to the formation of a hydrogen bond between N2 and N4 (N2···H–N4 = 2.823(3) Å). The O1–C7 and O2–C7 bond distances suggested a typical carboxyl group, while those of O3–C14 and O4–C14 indicate a carboxylate ion. The C–C bonds in the six- membered ring of DBA indicate a high level of aromaticity. In the crystal, units A and B formed a chain structure with hydrogen bonds between O1 and O3 (O1–H···O3 = 2.526(2) Å), as well as between N2 and N4.

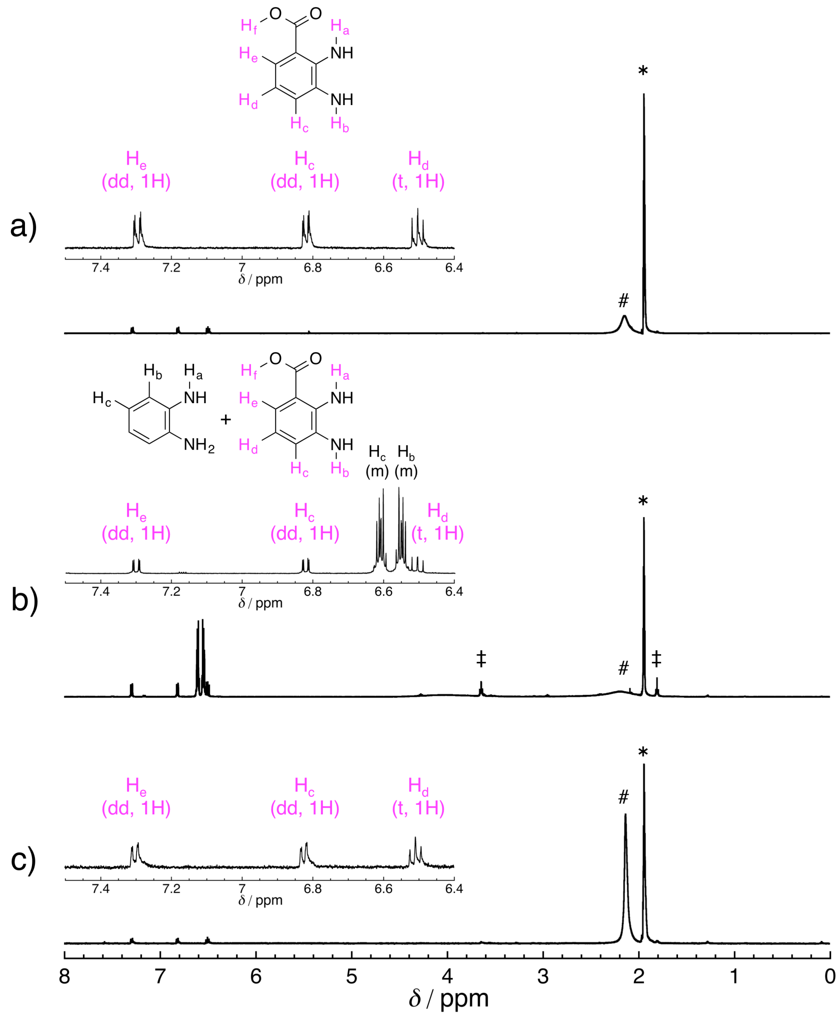

Supplementary Fig. 6 ^{1}H NMR (CD_3CN, 500 MHz) spectra of a) commercial DBA, b) the crude photochemical products of opda (6.0×10^{-3} M, 8 h), and c) the photochemical product of **1** (2.0×10^{-3} M, 8 h) after column chromatography at room temperature under CO_2. The insets show the magnified spectral region of 6.4–7.5 ppm. The symbols "*", "#", and "‡" indicate NMR signals from residual protons of CD_3CN, H_2O, and THF, respectively. Amino and carboxylic protons were not observed, probably due to signal broadening, which has previously been reported for the ^{1}H NMR spectral data of DBA in DMSO-d_6.[4-6]

In the ^{1}H NMR spectrum of commercial DBA, characteristic two sets of doublets and a triplet were observed at 6.51, 6.82, and 7.30 ppm, respectively (Supplementary Fig. 6a). The spectral fingerprints were almost identical to those of the newly emerged signals observed in the spectrum of the product formed by the photoreaction of opda or **1** under CO_2 (Supplementary Figs. 6b and c). The signals for the aromatic protons, H_c and H_e, of DBA were downfield shifted compared with those of opda, which suggests that the electron density on the aromatic ring was reduced due to the introduction of the carboxyl group.

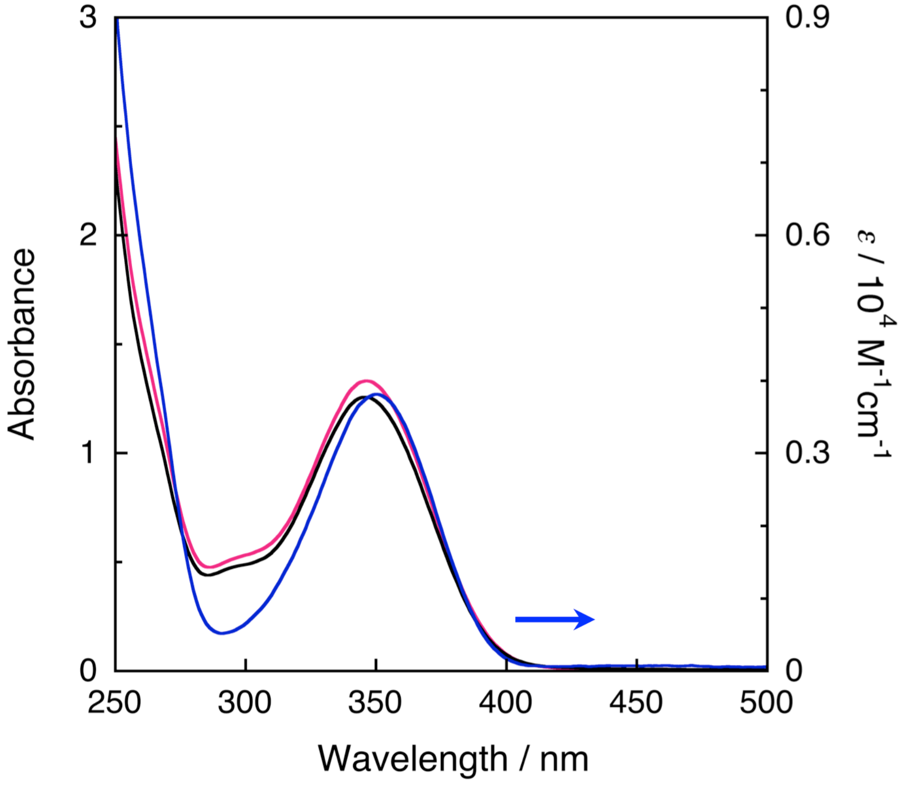

Supplementary Fig. 7 UV-Vis spectra of the photochemical products (λ_{ex} = 300±10 nm; 8 h; 63.9–66.9 mW) using *ex-situ-* (–) or *in-situ-* (–) prepared **1**, together with that of commercial DBA (–), under CO_2 in THF ($[Fe^{II}(H_2O)_6][ClO_4]_2$ = 2.0 × 10^{-3} M with 3 eq. of opda for *ex-situ-*, and 2.0 × 10^{-3} M for *in-situ*-prepared **1**).

The photochemical reaction of **1**, prepared *in-situ* by mixing $[Fe^{II}(H_2O)_6][ClO_4]_2$ and 3 eq. of opda (run 3 in Table 1), resulted in the formation of DBA in comparable 58.0% yield (Φ = 0.47%) to that obtained from *ex-situ*-prepared **1** (Supplementary Fig. 7 and run 1 in Table 1). This result suggests that there was no significant influence of the preparation method on the photochemical reactions.

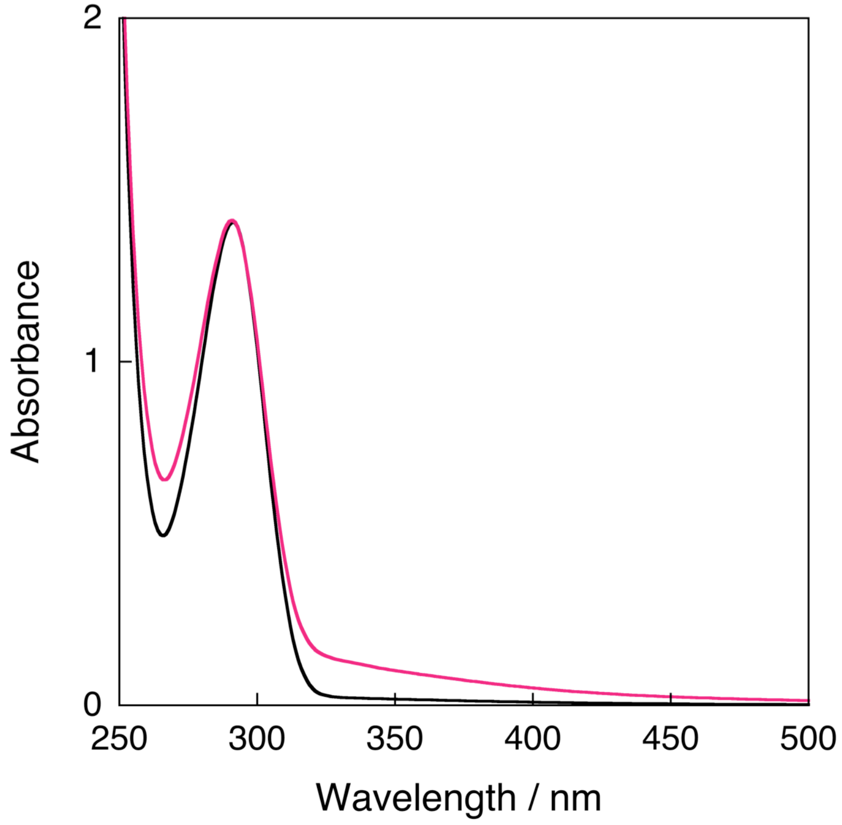

Supplementary Fig. 8 UV-Vis spectrum of the mixture of $[Fe^{II}(H_2O)_6][ClO_4]_2$ (2.0×10^{-3} M) with 3 eq. of aniline (–) and that obtained after photoirradiation (λ_{ex} = 289±10 nm; 8 h; 55.1 mW) (–).

The photoreaction using aniline (Supplementary Fig. 8 and run 5 in Table 1) was performed with Fe(II) under CO_2. The UV-Vis spectral fingerprints before and after the photoreaction were similar, indicating that aniline was inert towards the applied photo-irradiation. The different results of aniline compared to those of opda and/or **1** suggest that a bidentate coordinating interaction of the amino groups with Fe(II) or substituent effects derived from at least two amino groups are necessary for the observed photochemical carboxylation.

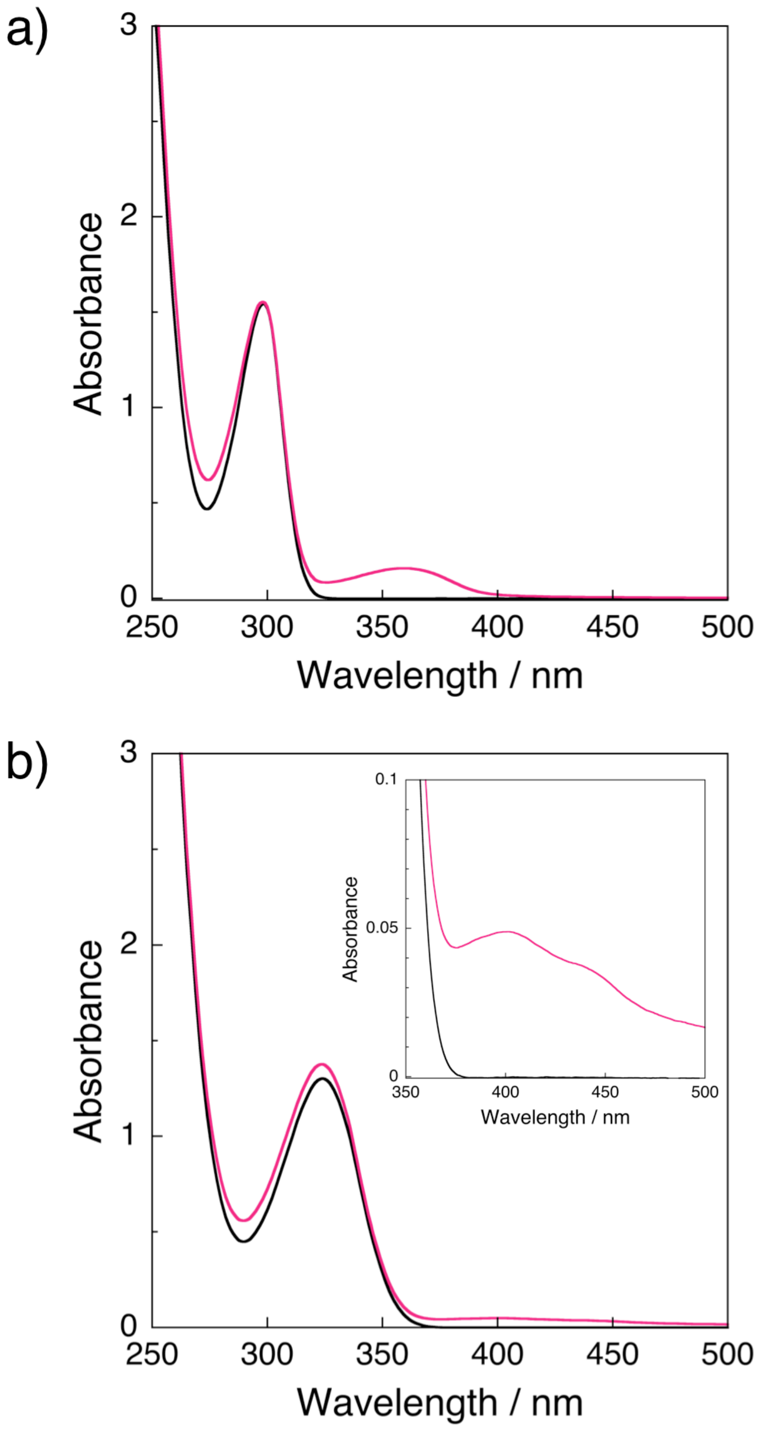

Supplementary Fig. 9 UV-Vis spectra of a) mpda and b) ppda in THF (6.0 × 10^{-3} M) before (–) and after photo-irradiation (λ_{ex} = 300±10 nm; 63.5–66.9 mW; 8 h) (–) under CO_2 at room temperature. The inset in b) shows the magnified spectrum (350-500 nm).

In the UV-Vis spectra of mpda and ppda in THF, the absorption maxima observed at 298 and 324 nm were tentatively assigned to π–π* transitions.

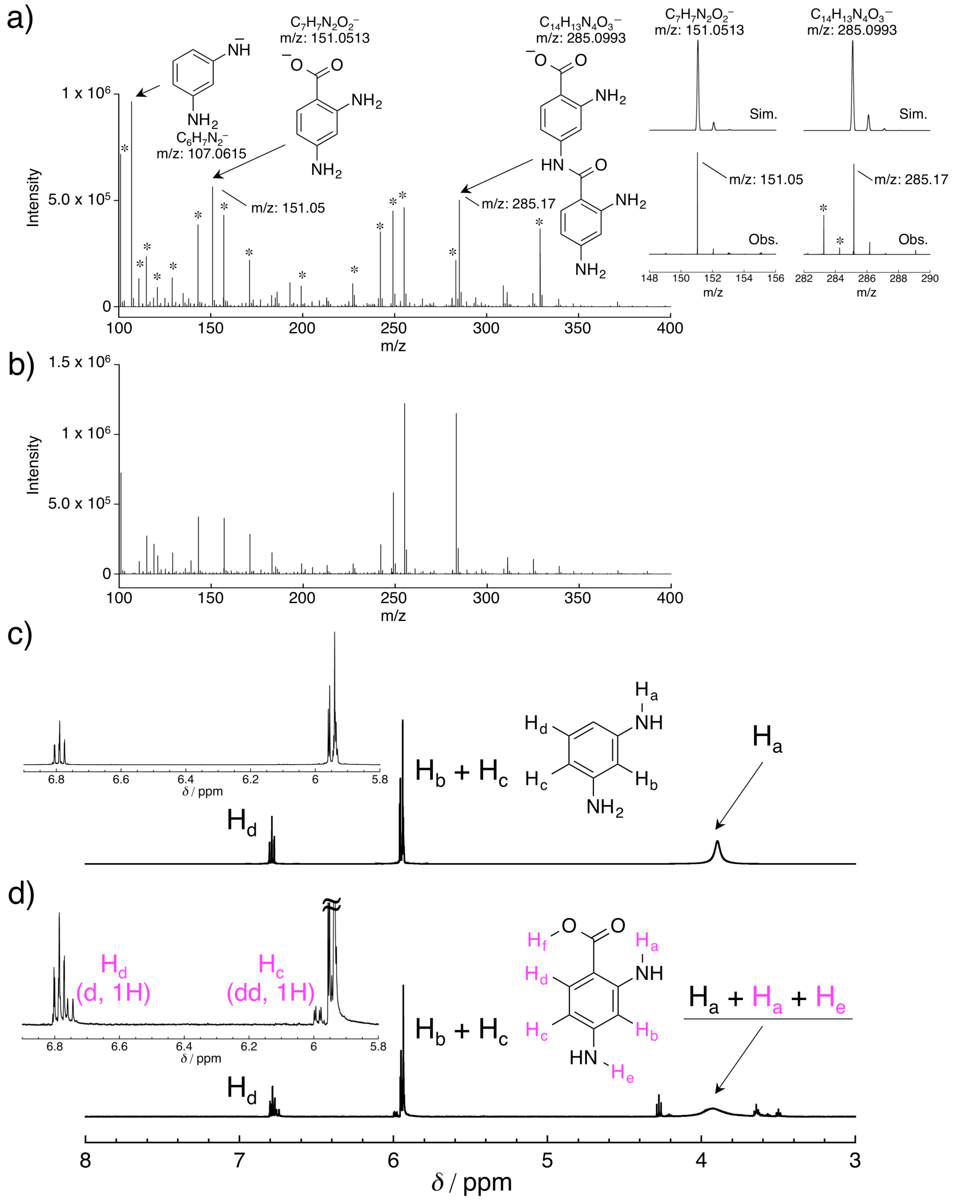

Supplementary Fig. 10 a) Negative-mode ESI-MS spectrum of the photochemical products of mpda (6.0×10^{-3} M) after irradiation (λ_{ex} = 300±10 nm; 8 h; 66.3 mW) in THF under CO_2 at room temperature. After the removal of THF, the sample was dissolved in MeOH and this solution was used for measurements. b) Background ESI-MS spectrum. Signals marked with * in a) can be attributed to be the background. ^{1}H NMR (CD_3CN, 500 MHz) spectra of c) pure mpda and d) the photochemical product of mpda. The insets show the magnified spectra in the region of 5.8–6.9 ppm for c) and d). The signals of the H_b and H_f protons of 2,4-diaminobenzoic acid could not be identified dur to overlapping with those of mpda.

In the ESI-MS spectrum of the crude sample, the signal at *m/z* 151.05 corresponds to the carboxylated product (Supplementary Fig. 10a). Based on the spectral pattern and molecular symmetry, the newly emerged signals observed in the ^{1}H NMR spectra of the crude product were tentatively assigned to 2,4-diaminobenzoic acids (Supplementary Fig. 10d).

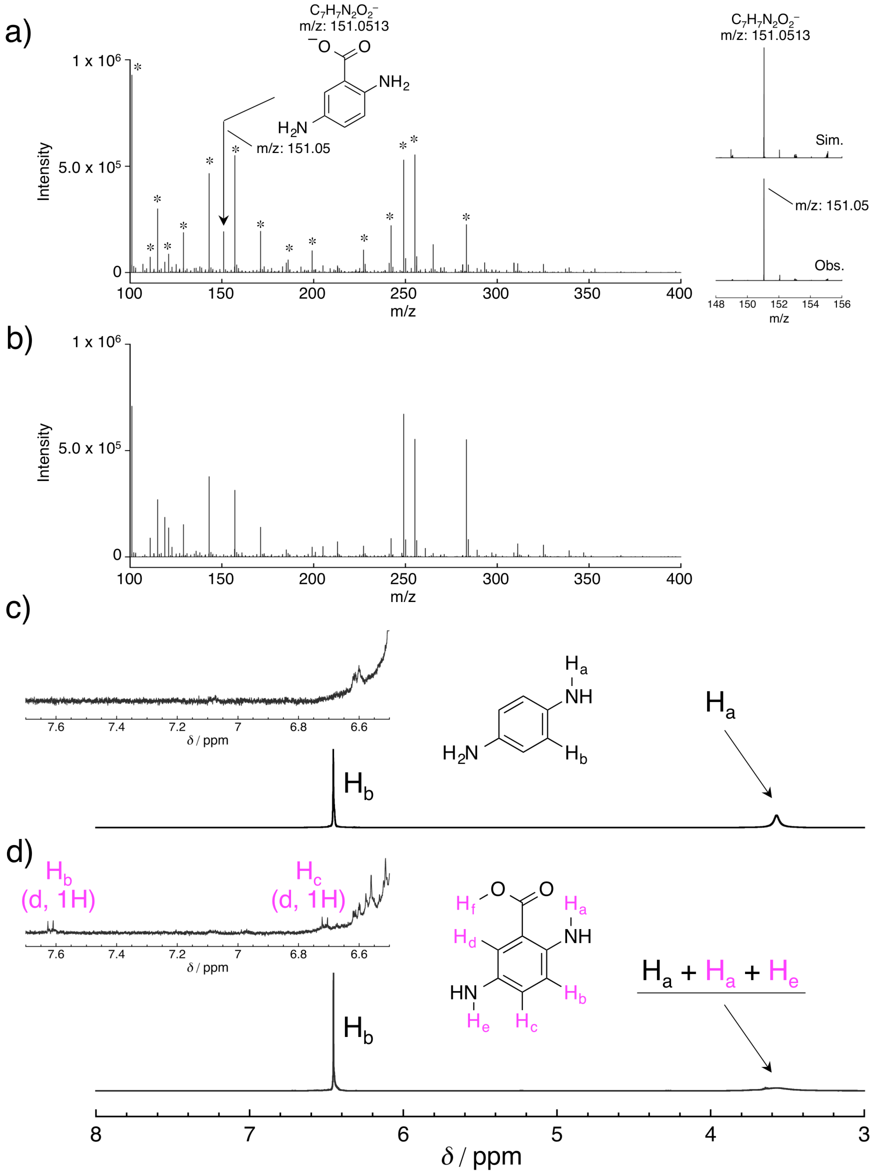

Supplementary Fig. 11 a) Negative-mode ESI-MS spectrum of the photochemical product of ppda (6.0 × 10^{-3} M) in THF after irradiation (λ_{ex} = 300±10 nm; 8 h; 63.5 mW) at room temperature under CO_2. After removal of the THF, the sample was dissolved in MeOH and this solution was used for the measurements. b) Background ESI-MS spectrum. Signals marked with * in a) are attributed to the background. ^{1}H NMR (CD_3CN, 500 MHz) spectra of c) pure ppda and d) the photochemical product of ppda. The insets show the magnified spectral region of 6.5–7.7 ppm for c) and d). The signals for the H_d and H_f protons of 2,5-diaminobenzoic acid could not be identified due to their low content and overlap with those of ppda.

In the ESI-MS spectrum of the crude sample, the signal at *m*/*z* 151.05 corresponds to the carboxylated product (Supplementary Fig. 11). Based on the spectral pattern and molecular symmetry, the newly emerged signals observed in the ^{1}H NMR spectra of the crude product were tentatively assigned to 2,5-diaminobenzoic acids (Supplementary Fig. 11d).

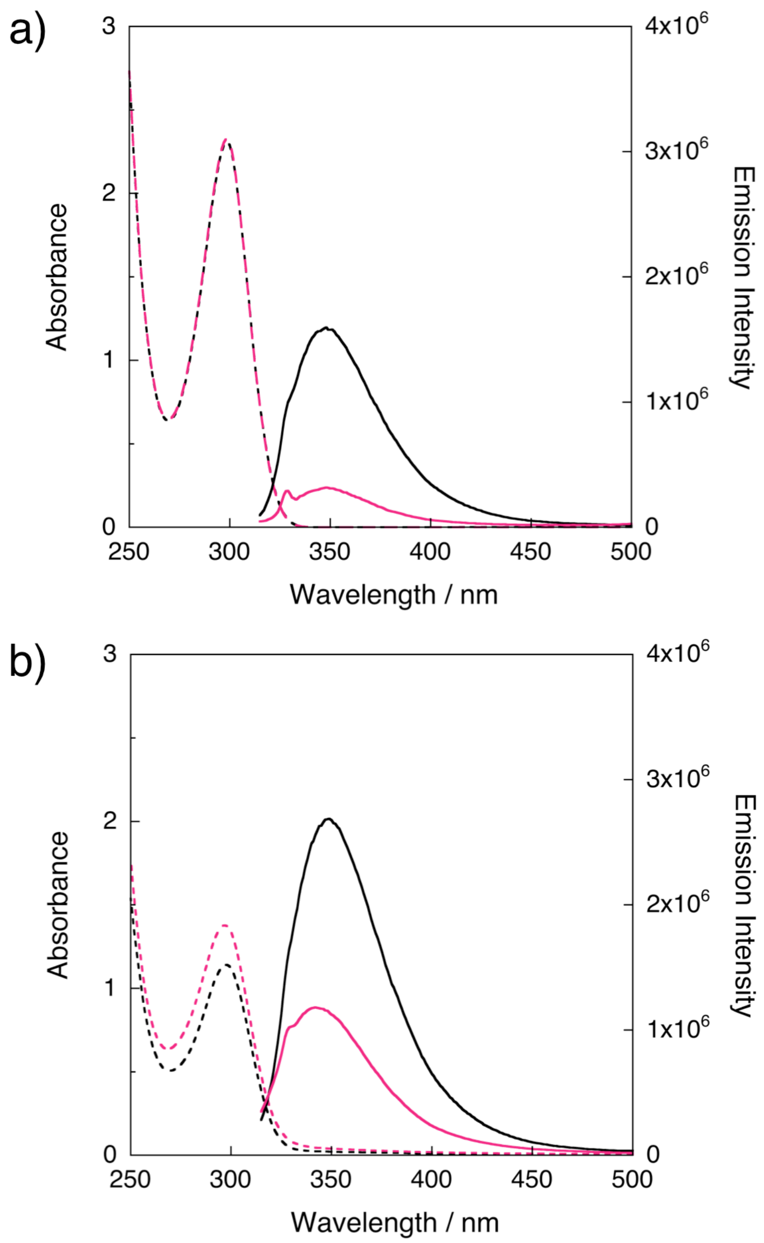

Supplementary Fig. 12 Emission spectra of a) opda (2.6×10^{-5} M) and b) **1** (1.3×10^{-5} M) in THF at room temperature under N_2 (–) or CO_2 (–) (λ_{ex} = 300 nm), as well as the absorption spectra of opda (6.0×10^{-3} M) and **1** (2.0×10^{-3} M) in THF at room temperature under N_2 (···) or CO_2 (···).

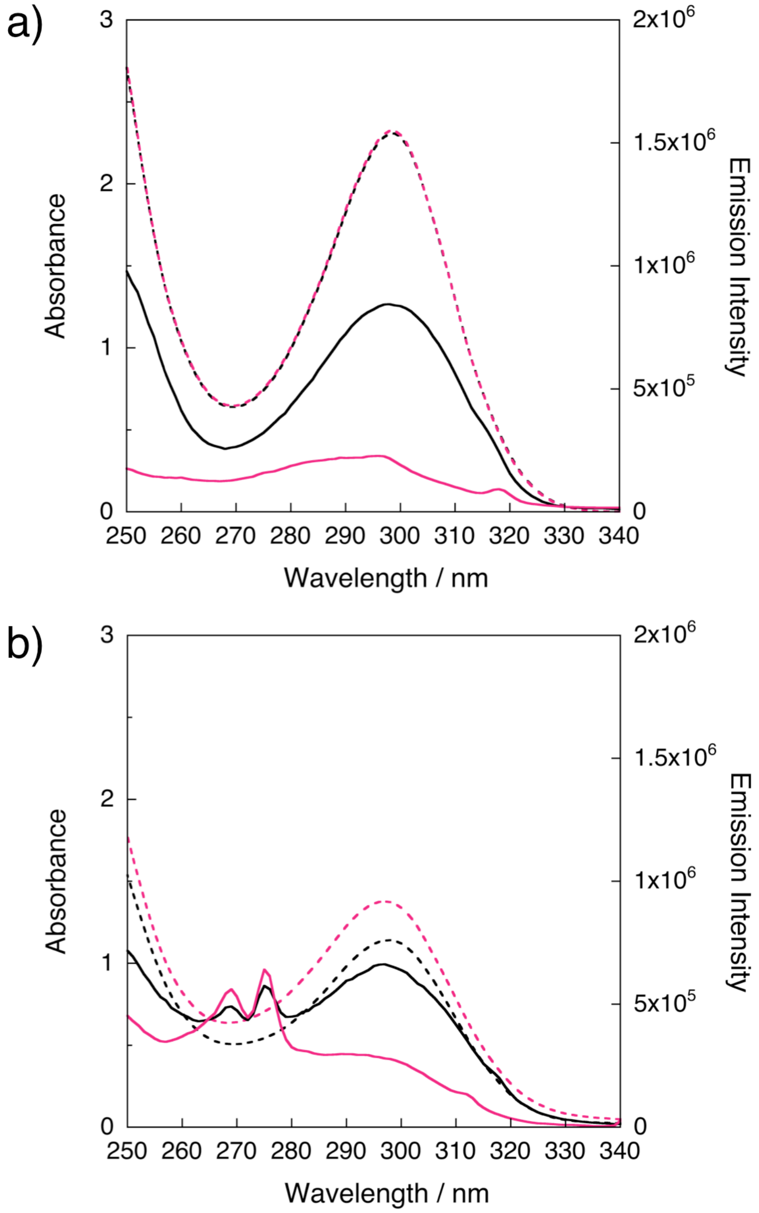

Supplementary Fig. 13 Excitation spectra of a) opda (2.6 × 10^{-5} M) and b) **1** (1.3 × 10^{-5} M) in THF at room temperature under N_2 (–) or CO_2 (–) (λ_{obs} = 350 nm), together with the absorption spectra of opda (6.0 × 10^{-3} M) and **1** (2.0 × 10^{-3} M) in THF at room temperature under N_2 (···) or CO_2 (···).

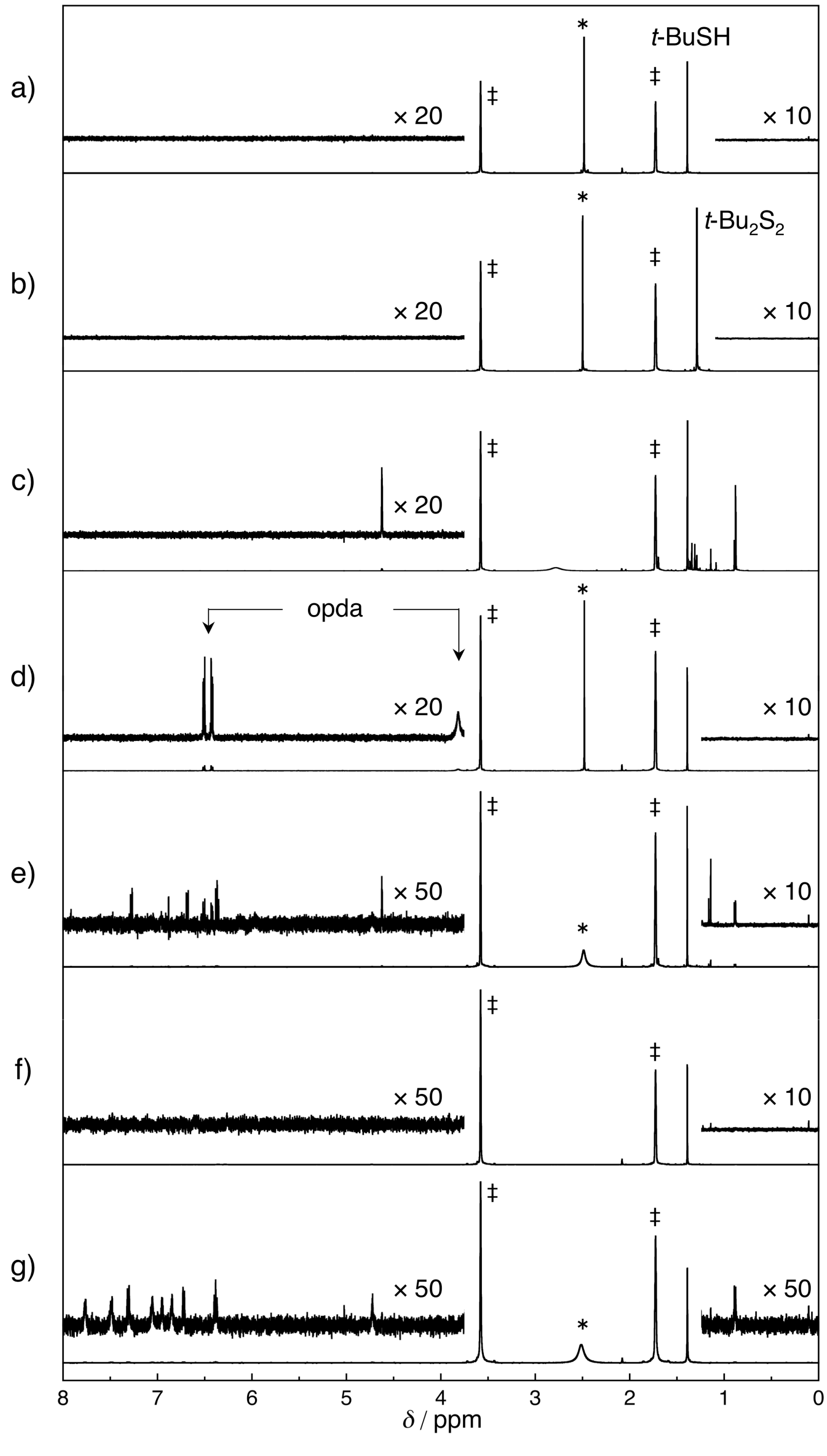

Supplementary Fig. 14 ^{1}H NMR (THF-d_8, 500 MHz) spectra (0.0-8.0 ppm) of a) *t*-BuSH, b) *t*-Bu_2S_2, c) *t*-Bu_2S_2 after photoirradiation (λ_{ex} = 300±10 nm; 64.5 mW; 3 h), d) *t*-BuSH with opda, e) *t*-BuSH after photoirradiation (λ_{ex} = 300±10 nm; 64.2 mW; 3 h) in the presence of opda, f) *t*-BuSH with **1**, and g) *t*-BuSH after photoirradiation (λ_{ex} = 300±10 nm; 64.5 mW; 3 h) in the presence of **1**, measured under CO_2 at room temperature. The symbols "‡" and "*" indicate the NMR signals from residual protons of THF-d_8 and H_2O, respectively.

Supplementary Method

Crystallographic data collection and structure refinement. Single-crystal X-ray diffraction analyses of **1** under CO_2 and DBA were carried out on a Rigaku VariMax diffractometer with Saturn equipment, using graphite-monochromated Mo-Kα radiation (λ = 0.71075 Å). Single crystals of suitable size and quality were selected under paraffin oil, mounted on a MicroMounts (MiTeGen), and cooled to 183 K (**1**) or 93 K (DBA) with a N_2 flow-type temperature controller. Molecular structures were solved by direct methods (SIR2004),[7] which allowed the successful location of all non-hydrogen atoms within the unit cell. All calculations were carried out using the CrystalStructure crystallography software package,[8] except for the refinement calculations, which were performed using SHELXL-97.[9] A summary of the crystallographic data for **1** and DBA is shown in Supplementary Tables 1 and 2, and complete structural details of DBA have been deposited at the Cambridge Crystallographic Data Centre (CCDC) under reference number CCDC-1826028.

Supplementary References

1. Matsumoto, T. *et al*. Nonprecious-metal-assisted photochemical hydrogen production from *ortho*-phenylenediamine. *J. Am. Chem. Soc*. **135,** 8646-8654, (2013).

2. Lai, T. F. & Marsh, R. E. The crystal structure of *p*-aminobenzoic acid. *Acta Crystallogr*. **22**, 885-893, (1967).

3. Athimoolam, S. & Natarajan, S. 4-Carboxyanilinium (*2R*,*3R*)-tartrate and a redetermination of the α-polymorph of 4-aminobenzoic acid. *Acta Crystallogr. Sect. C: Cryst. Struct. Commun.* **63**, o514-o517, (2007).

4. Lavigueur, C., Foster, E. J. & Williams, V. E. Self-Assembly of discotic mesogens in solution and in liquid crystalline phases: Effects of substituent position and hydrogen bonding. *J. Am. Chem. Soc.* **130**, 11791-11800, (2008).

5. Schmidt, A., Shilabin, A. G. & Nieger, M. On benzo[*b*][1,4]diazepinium-olates, -thiolates and -carboxylates as anti-Huckel mesomeric betaines. *Org. Biomol. Chem.* **1**, 4342-4350, (2003).

6. Hansen, M. *et al.* Synthesis and pharmacological evaluation of *N*-benzyl substituted 4-bromo-2,5-dimethoxyphenethylamines as 5-$HT_{2A/2C}$ partial agonists. *Biorg. Med. Chem.* **23**, 3933-3937, (2015).

7. Burla, M. C. *et al*. SIR2004: an improved tool for crystal structure determination and refinement. *J. Appl. Crystallogr*. **38,** 381-388, (2005).

8. CrystalStructure 3.8.2, Crystal Structure Analysis Package; Rigaku and Rigaku/MSC: The Woodlands, TX, 2000−2006.

9. Sheldrick, G. SHELX-97 Program for crystal structure solution and the refinement of crystal structures, Institut für Anorganische Chemie der Universität Göttingen, Tammanstrasse 4, D-3400 Göttingen, Germany, 1997.
