# Supplementary material for: Direct Photochemical C–H Carboxylation of Aromatic Diamines with CO2 under Electron-Donor- and Base-free Conditions
Source: Sci Rep. 2018 Oct 2;8:14623. doi: 10.1038/s41598-018-33060-3 (PMC6168591; doi:10.1038/s41598-018-33060-3)

checkCIF/PLATON report

Structure factors have been supplied for datablock(s) DBA

THIS REPORT IS FOR GUIDANCE ONLY. IF USED AS PART OF A REVIEW PROCEDURE FOR PUBLICATION, IT SHOULD NOT REPLACE THE EXPERTISE OF AN EXPERIENCED CRYSTALLOGRAPHIC REFEREE.

No syntax errors found. CIF dictionary Interpreting this report

Datablock: DBA

```
Bond precision:  C-C = 0.0030 A          Wavelength=0.71075

Cell:            a=8.646(5)     b=11.344(6)         c=13.893(8)
                 alpha=90       beta=99.209(10)     gamma=90
Temperature:     93 K

                Calculated                Reported
Volume          1345.1(13)                1345.1(13)
Space group     P 21/n                    P 1 21/n 1
Hall group      -P 2yn                    -P 2yn
Moiety formula  C14 H16 N4 O4             C7 H8 N2 O2
Sum formula     C14 H16 N4 O4             C7 H8 N2 O2
Mr              304.31                    152.15
Dx,g cm-3       1.503                     1.503
Z               4                         8
Mu (mm-1)       0.113                     0.113
F000            640.0                     640.0
F000'           640.31
h,k,lmax        11,14,18                  11,14,17
Nref            3075                      3045
Tmin,Tmax       0.992,0.995               0.868,0.996
Tmin'           0.981

Correction method= # Reported T Limits: Tmin=0.868 Tmax=0.996
AbsCorr = MULTI-SCAN

Data completeness= 0.990          Theta(max)= 27.443

R(reflections)= 0.0627( 1843)     wR2(reflections)= 0.1790( 3045)

S = 1.013                  Npar= 239
```

The following ALERTS were generated. Each ALERT has the format
test-name_ALERT_alert-type_alert-level.
Click on the hyperlinks for more details of the test.

Alert level B

```
PLAT482_ALERT_4_B Small D-H..A Angle Rep for N2       ..N1              87.20 Degree
```

Alert level C

```
PLAT303_ALERT_2_C Full Occupancy Atom H16       with # Connections       2.00 Check
PLAT353_ALERT_3_C Long   N-H (N0.87,N1.01A)  N4       - H8       .       1.04 Ang.
PLAT772_ALERT_2_C Suspect O-H Bond in CIF: O3       --H16       ..       1.36 Ang.
PLAT906_ALERT_3_C Large K Value in the Analysis of Variance ......     16.932 Check
PLAT906_ALERT_3_C Large K Value in the Analysis of Variance ......      3.350 Check
PLAT911_ALERT_3_C Missing FCF Refl Between Thmin & STh/L=    0.600          6 Report
```

Alert level G

```
PLAT042_ALERT_1_G Calc. and Reported MoietyFormula Strings  Differ     Please Check
PLAT045_ALERT_1_G Calculated and Reported Z Differ by a Factor ...       0.50 Check
PLAT112_ALERT_2_G ADDSYM Detects New (Pseudo) Symm. Elem       sub         90 %Fit
PLAT882_ALERT_1_G No Datum for _diffrn_reflns_av_unetI/netI ......     Please Do !
PLAT910_ALERT_3_G Missing # of FCF Reflection(s) Below Theta(Min).          4 Note
PLAT912_ALERT_4_G Missing # of FCF Reflections Above STh/L=  0.600         19 Note
PLAT913_ALERT_3_G Missing # of Very Strong Reflections in FCF ....          1 Note
PLAT978_ALERT_2_G Number C-C Bonds with Positive Residual Density.          2 Info
```

---

0 **ALERT level A** = Most likely a serious problem - resolve or explain
1 **ALERT level B** = A potentially serious problem, consider carefully
6 **ALERT level C** = Check. Ensure it is not caused by an omission or oversight
8 **ALERT level G** = General information/check it is not something unexpected

3 ALERT type 1 CIF construction/syntax error, inconsistent or missing data
4 ALERT type 2 Indicator that the structure model may be wrong or deficient
6 ALERT type 3 Indicator that the structure quality may be low
2 ALERT type 4 Improvement, methodology, query or suggestion
0 ALERT type 5 Informative message, check

It is advisable to attempt to resolve as many as possible of the alerts in all categories. Often the minor alerts point to easily fixed oversights, errors and omissions in your CIF or refinement strategy, so attention to these fine details can be worthwhile. In order to resolve some of the more serious problems it may be necessary to carry out additional measurements or structure refinements. However, the purpose of your study may justify the reported deviations and the more serious of these should normally be commented upon in the discussion or experimental section of a paper or in the "special_details" fields of the CIF. checkCIF was carefully designed to identify outliers and unusual parameters, but every test has its limitations and alerts that are not important in a particular case may appear. Conversely, the absence of alerts does not guarantee there are no aspects of the results needing attention. It is up to the individual to critically assess their own results and, if necessary, seek expert advice.

Publication of your CIF in IUCr journals

A basic structural check has been run on your CIF. These basic checks will be run on all CIFs submitted for publication in IUCr journals (*Acta Crystallographica*, *Journal of Applied Crystallography*, *Journal of Synchrotron Radiation*); however, if you intend to submit to *Acta Crystallographica Section C* or *E* or *IUCrData*, you should make sure that full publication checks are run on the final version of your CIF prior to submission.

Publication of your CIF in other journals

Please refer to the *Notes for Authors* of the relevant journal for any special instructions relating to CIF submission.

PLATON version of 30/01/2018; check.def file version of 30/01/2018

Datablock DBA - ellipsoid plot

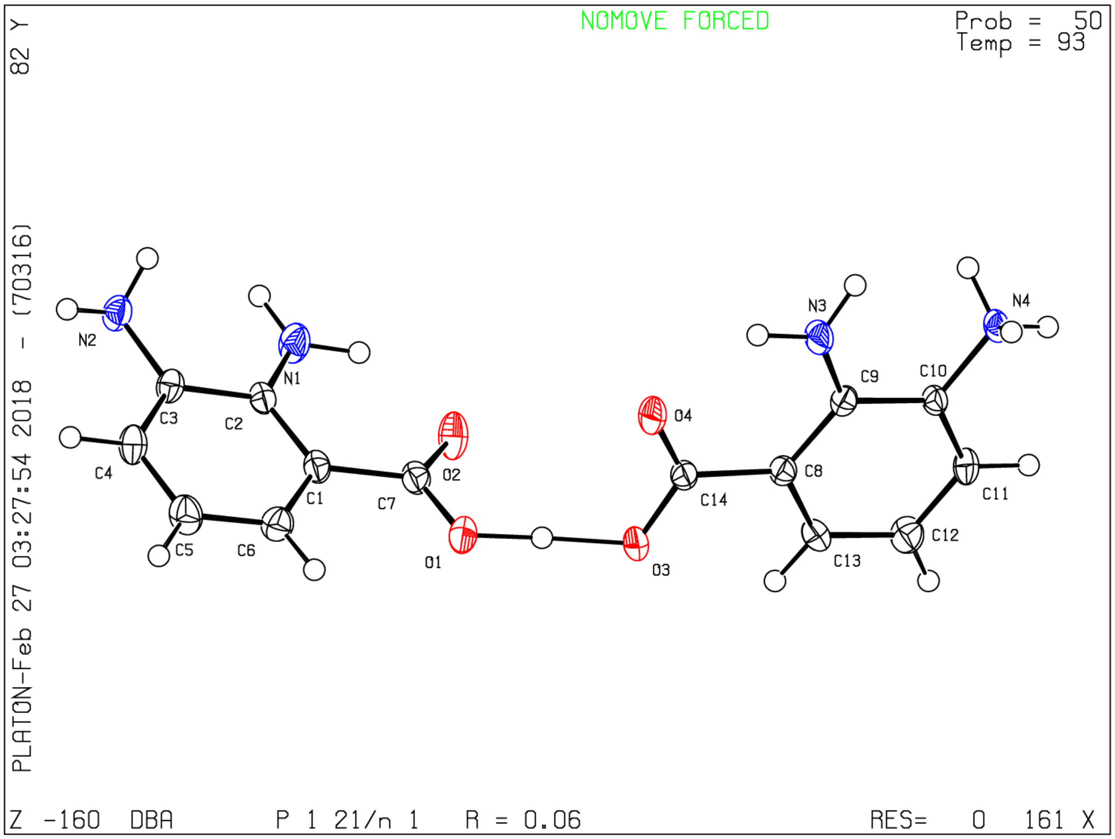

Supplement: Supplementary file 3 — Supplementary Dataset 2 [file 41598_2018_33060_MOESM3_ESM.pdf]
